# Comprehensive profiling of the chemical constituents in Dayuanyin decoction using UPLC-QTOF-MS combined with molecular networking

**DOI:** 10.1080/13880209.2024.2354341

**Published:** 2024-05-29

**Authors:** Jing Peng, Chengyu Ge, Kaiqi Shang, Shao Liu, Yueping Jiang

**Affiliations:** a Department of Pharmacy, Xiangya Hospital, Central South University, Changsha, China; b Institute for Rational and Safe Medication Practices, National Clinical Research Center for Geriatric Disorders, Xiangya Hospital, Central South University, Changsha, China; c College of Pharmacy, Changsha Medical University, Changsha, Hunan, China

**Keywords:** UPLC-QTOF-MS, Dayuanyin decoction, integrated strategy, molecular networking

## Abstract

**Context:**

Dayuanyin decoction is a traditional Chinese medicine formulation that is commonly used in modern clinical practice to treat viral infections such as viral pneumonia, viral fever, influenza, and hepatitis. Although the usage rate of Dayuanyin decoction is gradually increasing in clinical practice, its pharmacological constituents are still unclear.

**Objective:**

This study comprehensively characterized the chemical constituents in Dayuanyin decoction using ultra-performance liquid chromatography quadrupole time-of-flight mass spectrometry (UPLC-QTOF-MS) and molecular networking.

**Materials and methods:**

The overall strategy involved retrieving structural information, such as fragment ions and precursor ion masses, from self-built databases to identify the target constituents of the Dayuanyin decoction extract. The identification of non-targeted constituents was achieved by analyzing different categories, fragment pathways, mass spectrometry data, and the relationships between clusters of structures in molecular networking. Unannotated constituents were inferred from secondary mass spectrometry similarity and molecular weight differences and annotated constituents in the same constituent cluster. A few predicted constituents were selected and validated by comparing them to reference standards under identical mass spectrometry conditions.

**Results:**

This study preliminarily identified 216 constituents, including flavonoids, amino acids, alkaloids, triterpenes, steroidal saponins, phenylpropanoids, and other constituents.

**Conclusions:**

This integrated strategy using UPLC-QTOF-MS and molecular networking lays the foundation for clinical research on pharmacologically active substances in Dayuanyin decoction and could be popularized for the comprehensive profiling of chemical constituents of other traditional Chinese medicines.

## Introduction

Dayuanyin decoction was first documented in the ‘Plague Theory’ by Wu Youke during the Ming Dynasty. According to the ‘Plague Theory’, Dayuanyin decoction was originally designed to treat the early stages of the plague and was believed to open up membranes, remove turbidity, clear heat, and detoxify the body (Hu et al. 2020; Ruan et al. 2020; Jin et al. 2020). A previous study found that lamivudine and Dayuanyin decoction could inhibit the secretion of hepatitis B surface antigen (HBsAg) in the culture supernatant of HepG 2.2.15 cells at 72 h and 144 h, showing significant differences compared to the control group (*p* < 0.05, *p* < 0.01, respectively). This indicates a significant anti-HBV effect *in vitro* (Wang et al. 2012). Ren (2017) reported that the water extract, the supernatant of the water-extracted alcohol precipitation of Dayuanyin decoction, and other constituents could alleviate pulmonary edema in mice with acute lung injury induced by lipopolysaccharide, reducing lung tissue damage. Experiments by the author showed that Dayuanyin decoction had a significant antipyretic effect, which was related to the decreases in interleukin-6 (IL-6) and tumor necrosis factor-alpha (TNF-α) levels in serum and decreases in myeloperoxidase activity in liver tissue (Ren et al. 2015). Modern physicians widely use Dayuanyin decoction to treat a variety of systemic conditions, such as fever, respiratory, digestive, urinary, and endocrine diseases, as well as skin and pediatric disorders. More importantly, Dayuanyin decoction played a significant role in the treatment of Corona Virus Disease 2019 (COVID-19) pandemic (Ding et al. 2020). Therefore, Dayuanyin decoction is gaining increasing attention among medical professionals and researchers.

The chemical constituents contained in Dayuanyin decoction mainly include alkaloids, flavonoids, terpenes, saponins, phenylpropanoids, and essential oils (Xiao et al. 2023). Currently, few studies have analyzed the constituents of a water extract of Dayuanyin decoction, as most have focused on methanol and ethanol extracts. The water extraction method is generally used for the administration of Dayuanyin decoction, making it necessary to research its water-soluble constituents. Constituent research has traditionally been conducted by building databases by collecting ion information from published studies and using that information to identify constituents. Although this method can provide accurate results, it is inefficient. In contrast, the present study used a rapid detection method, combining Agilent′ s internal database with constituent similarity provided by molecular networking. This method relied on software analysis to infer unknown constituents related to identified constituents, allowing for the quick identification of constituents in the mixture. Therefore, this method was employed to analyze the constituents in a water extract of Dayuanyin decoction and summarize the fragmentation patterns of various constituent classes.

Global Natural Product Social (GNPS) molecular networking (Nothias et al. 2020) is a public data analysis platform established by annotating new spectra in the existing multiple mass spectrometry databases. It includes 272 public datasets and 84 million tandem mass spectroscopy (MS/MS) spectra, providing an automated molecular networking (MN) tool for analyzing MS/MS datasets (Wang et al. 2016). MN utilizes mass spectrometry information and fragmentation pathways to group multiple unannotated constituents in complex matrices with known constituents based on spectral similarity. MN is formed by connecting molecules with similar fragmentation spectra to study their properties (Zhao et al. 2022; Beniddir et al. 2021). MN has been successfully applied to the discovery of novel natural products, microorganisms, fungi, and marine organisms. MN combined with UPLC-QTOF-MS is also widely used for analyzing fungal metabolites and specific constituents in traditional Chinese medicinal plants (Chen et al. 2021; Messaili et al. 2020).

UPLC-QTOF-MS has advantages that include high resolution, high sensitivity, and high accuracy, making it an indispensable method for exploring chemical substances in traditional Chinese medicine (Liu et al. 2013). However, the processing of large amounts of data obtained from MS/MS is both time-consuming and complex. Therefore, this study employed a high-throughput and highly sensitive UPLC-QTOF-MS combined with the MN method to rapidly elucidate the structures of non-targeted and complex constituents in the traditional Chinese medicine Dayuanyin decoction. Additionally, an internal database was utilized to identify known and unknown constituents in the Dayuanyin decoction based on constituent similarity, achieving the comprehensive identification of the chemical constituents present in the Dayuanyin decoction formulation.

## Materials and methods

### Chemicals and reagents

Acetonitrile (LC-MS-grade) and methanol (HPLC-grade) were purchased from Merck (Darmstadt, Germany). LC-MS-grade formic acid was purchased from Sigma-Aldrich (St. Louis, MO, USA; cat. no. 197147).

### Plant material

*Areca catechu* L. (Arecaceae) (origin: Hainan, batch number 22011905); *Anemarrhena asphodeloides* Bge. (Liliaceae) (origin: Henan, batch number 22081104); *Glycyrrhiza uralensis* Fisch. (Fabaceae) (origin: Inner Mongolia, batch number 22062406); *Scutellaria baicalensis* Georgi (Lamiaceae) (origin: Shanxi, batch number 22032405); *Magnoliae officinalis* Rehd. et Wils. (Magnoliaceae) (origin: Sichuan, batch number 22092307); and *Amommum tsao-ko* Crevost et Lemarie (Zingiberaceae) (origin: Guangxi, batch number 21041507) were purchased from Hunan Zhenxing Traditional Chinese Medicine Co., Ltd (Hunan, Changsha, China). *Paeonia lactiflora* Pall. (Paeoniaceae) (origin: Zhejiang, batch number 20220802) was purchased from Hunan Jinxiang Pharmaceutical Co., Ltd. These herbal medicines were all purchased on October 9, 2022. All the above medicinal materials were authenticated by Professor Liu Shao from the Pharmacy Department of Xiangya Hospital, Central South University.

### Sample preparation

A 180 g sample of *Areca catechu* (seeds), 90 g of *Magnoliae officinalis* (bark), 45 g of *Amommum tsao-ko* (fruit), 90 g of *Anemarrhena asphodeloides* (rhizoma), 90 g of *Paeonia lactiflora* (roots), 90 g of *Scutellaria baicalensis* (roots), and 45 g of *Glycyrrhiza uralensis* (roots and rhizoma) were mixed in a distillation flask. Five times the total weight of water was added for soaking for 30 min. Reflux extraction was performed for 1 h (timing was started after boiling). After filtration, 8 times its water weight was added to the residue for another 1 h of extraction, and it was filtered again.

### Framework of the integrated strategy

**Figure 1.**
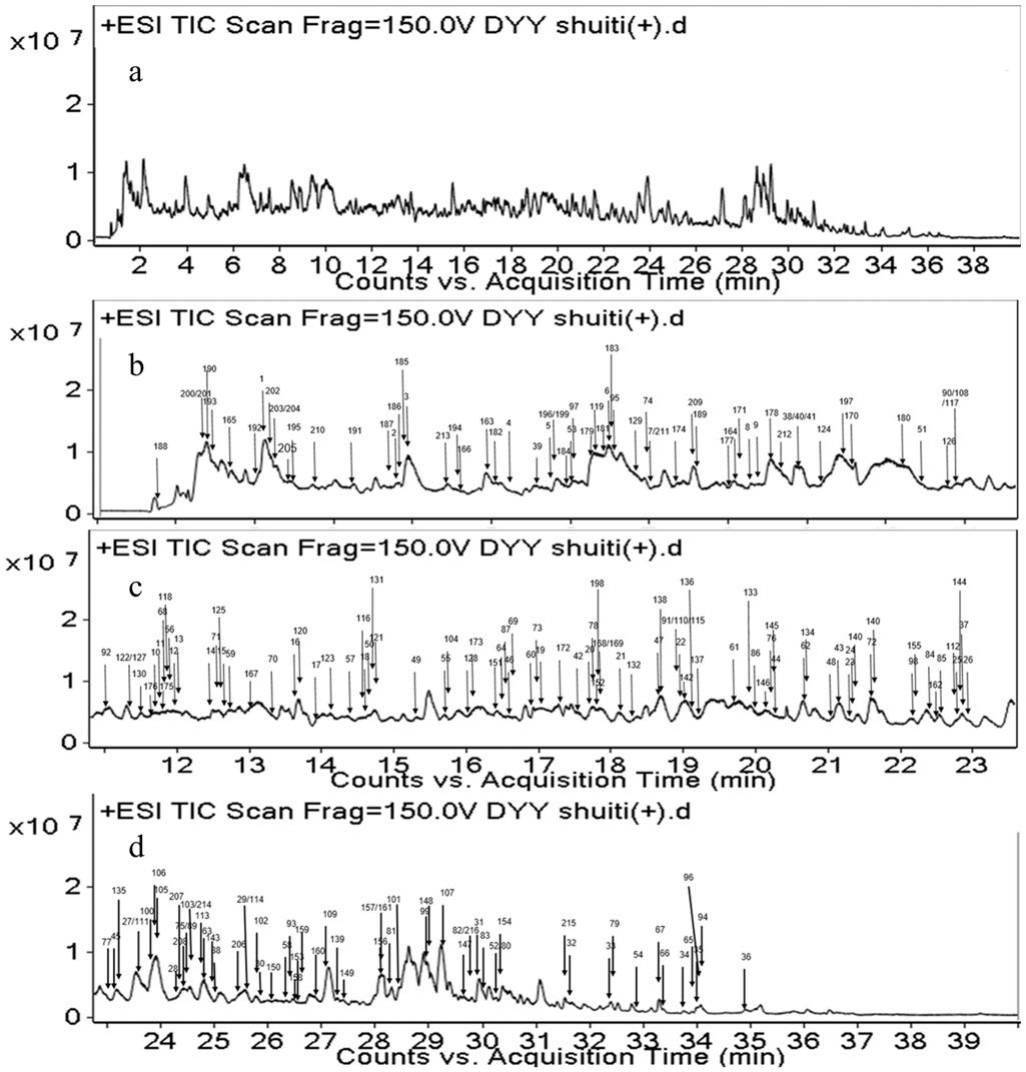
Total ion chromatogram of Dayuanyin decoction water extract in the positive ion mode (a). TIC of water extract of Dayuanyin decoction in the positive ion mode for 0–11 min, 11–23 min, and 23–40 min (b, c, and d).

**Figure 2.**
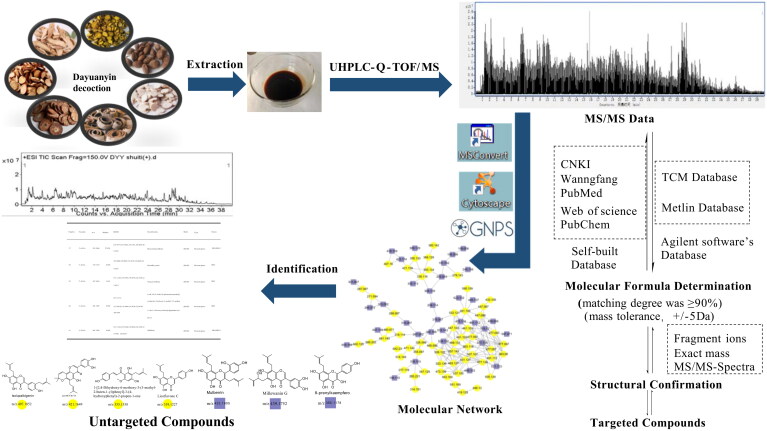
Dayuanyin decoction constituent identification strategy.

The purpose of this study was to preliminarily determine the chemical constituents in Dayuanyin decoction. The total ion flow chart obtained by mass spectrometry and the constituent numbers indicating segmented annotations are shown in Figure 1. A strategy of UPLC-QTOF-MS combined with GNPS molecular networking was used for the targeted screening of known constituents to achieve a more efficient identification method. MN was used for the structural classification of non-target tissues based on MS/MS spectral similarity. Starting from the nodes of known constituents, adjacent unreported constituents in the visualized network were determined through detailed analysis of MS/MS spectra and online database retrieval. The specific strategic process is illustrated in Figure 2.

First, target constituents were identified by compiling and summarizing the mass spectrometry information of relevant constituents in Dayuanyin decoction by collating data from journal platforms, such as the Web of Science (https://webofscience.clarivate.cn), the National Library of Medicine (https://pubmed.ncbi.nlm.nih.gov), the China National Knowledge Infrastructure (https://www.cnki.net/), and the Wanfang Data Knowledge Service Platform (https://www.wanfangdata.com.cn/). The data retrieved included constituent names, relative molecular mass, ion modes, parent ion *m/z* values, and MS/MS spectrometry fragments to establish a constituent database. Second, the Agilent MassHunter Qualitative Analysis B08.00 software′ s library matching function was utilized to match the mass spectrometry data of the Dayuanyin extract with the traditional Chinese medicine (TCM) and Metlin databases embedded in the software. Constituents with scores of ≥80 were selected. Finally, the matched constituents were compared with the fragment ions in the self-built database to identify the corresponding constituents.

The identification of non-target constituents was achieved by GNPS molecular networking. The identification of unannotated constituent nodes in the formed constituent clusters was based on the similarity of mass spectrometry features to constituents of the same type. The following steps were followed: First the elemental composition of the unannotated points was automatically deduced using the formula predictor in Qualitative Navigator B.08.00 software. The elements carbon (C), hydrogen (H), oxygen (O), nitrogen (N), and sulfur (S) were selected to calculate the elemental composition of the constituents. Only the highest-scoring and reasonable combinations were considered. In the second step, the predicted molecular formula was input into SciFinder to search for possible constituents. If a match was found, the structure of the unknown constituents could be determined based on the structures of the annotated points in the cluster, fragmentation patterns, and the differences in fragment ions that may exist between possible constituents. In the third step, Cytoscape 3.9.2 was used to visualize and edit the entire molecular networking dataset. These steps enabled the identification of unknown constituent structures within the MN results.

### UPLC-QTOF-MS analysis

The chromatographic evaluation was performed using an Agilent 1290 series UPLC system (Agilent Corp., Santa Clara, CA, USA) equipped with a binary pump, micro degasser, autosampler, and temperature-controlled column compartment. The sample was separated on an Agilent Eclipse C Plus C18 column (1.8 μm, 2.1 × 100 mm). The mobile phase was composed of two solvents (A and B). The mobile phase (A) was aqueous formic acid (0.1%, v/v), and the mobile phase (B) was acetonitrile. The following mobile phase gradient was used: 0–25 min: 5-35% B; 25–35 min: 35–95% B; and 35–40 min: 95% B. A positive ionization mode was employed in this experiment to ionize the constituents of the Dayuanyin decoction extract. The flow rate was 0.3 mL/min, the injection volume was 15 μL, and the column temperature was set at 40 °C.

Then, the analytes were passed through an Agilent 6545 A Q-TOF mass spectrometer (Agilent Corp.) equipped with an electrospray ionization (ESI) interface. The operating parameters were as follows. The drying N_2_ gas flow rate was 8 L/min, the temperature was 320 °C, the nebulizer was set at 35 PSI, the capillary energy was set to 3500 V, the MS/MS data acquisition mode was set to auto MS/MS, the max precursor per cycle was 5, and the acquisition time was 200 ms/spectrum. The samples were analyzed in the positive ion mode, and mass spectra data were recorded across a range of *m/z* 100–1700. Reference masses of 121.0509 (purine) and 922.0098 (HP-0921) were utilized for the internal mass calibration during runs in the positive ion mode. Fixed collision energies of 10.00, 20.00, and 40.00 V were chosen at a scan rate of 4.0 spectra/s using auto MS/MS data acquisition with a medium MS/MS isolation width.

### Molecular networking analysis

The original MS/MS spectral data were converted into an mzML format that contained all of the analysis information. Then, the mzML format file was uploaded to the client using FileZilla software and imported into the GNPS database for analysis (Chen et al. 2021; Rodrigues et al. 2022). Finally, based on the matched results, the MS/MS spectra were compared for similarity, identical ion fragments, and neutral losses to identify suitable constituents. The optimum parameters were as follows: parent mass and fragment tolerance of 0.02 Da, cosine score of ≥0.7, matched peaks ≥4, maximum connected constituent size of 100, minimum cluster size of 1, and no run MSCluster. Lastly, the results were downloaded and saved, and the GNPS results were visualized using Cytoscape 3.9.2 software [https://cytoscape.org]. The identification of the constituents was supported by GNPS spectral libraries. The MS/MS spectra of the Dayuanyin decoction constituents were compared to the MS/MS spectra of the constituents contained in the GNPS library platform using the following parameters: a minimum number of library search-matched peaks of 4, a score threshold of 0.7, and a maximum analog search mass difference of 100.

## Results

### Targeted identification results

The UPLC-QTOF-MS analysis of the water extract of Dayuanyin decoction was performed, with a detection time of 40 min. The error range was controlled within 5 ppm, and the matching degree was ≥90%. A total of 36 target constituents were identified in the extract through fragment ion information, TCM (https://www.tcmdatabase.com/), and Metlin databases (https://metlin.scripps.edu/landing_page.php?pgcontent=mainPage), which were mainly flavonoids and alkaloids. Specific information, including the constituent′ s names, retention times, formulas, precursor ions, and fragment ions, appears in Table 1.

**Table 1.** Characterisation of constituents in water extract of dayuanyin decoction by UPLC-QTOF-MS and MS/MS analysis in positive ion mode.

Number	t_R_(min)	m/z	Identification	MSMS	Formula	Mode
_1_	2.141	166.086	Phenylalanine	149.0597,120.0812,103.0545	C_9_H_11_NO_2_	[M + H]+
2	3.892	146.0605	Indole-4-carboxaldehyde	128.0488,118.0649	C_9_H_7_NO	[M + H]+
3	3.981	205.098	3-Amino-2-naphthoic acid	188.0705,146.0599,118.0646	C_11_H_9_NO_2_	[M + H]+
4	5.241	423.0914	Mangiferin	369.0586,339.0491,327.0496,303.0502,273.0390	C_19_H_18_O_11_	[M + H]+
5	5.716	579.1491	Procyanidin B2	427.0994,291.0838,271.0571,127.0376	C_30_H_26_O_12_	[M + H]+
6	6.477	314.1756	Codeine methyl ether	269.1178,237.0900,175.0751,137.0596,107.0495	C_19_H_23_NO_3_	[M + H]+
7	7.064	465.1007	Wedelolactone	303.0495,145.0481	C_21_H_20_O_12_	[M + H]+
8	8.331	291.0866	(-)-Epicatechin	179.0686,147.0434,139.0388,123.0436,	C_15_H_14_O_6_	[M + H]+
9	8.394	303.0498	Herbacetin	181.0487,133.0272,121.0435	C_15_H_10_O_7_	[M + H]+
10	11.656	328.1556	6-Monoacetylmorphine	297.1108,265.0845,205.0639,177.0535	C_19_H_21_NO_4_	[M + H]+
11	11.686	342.1687	Naltrexone	324.1596,282.0877,267.0645	C_20_H_23_NO_4_	[M + H]+
12	11.982	273.0729	Naringenin	153.0169,147.0426,119.0483	C_15_H_12_O_5_	[M + H]+
13	12.050	447.1260	Isoprunetin 7-*O*-β-D-glucopyranoside	285.0747,270.0500,253.0469,225.0523,137.0225	C_22_H_22_O_10_	[M + H]+
14	12.454	433.1122	Vitexin	415.1025,397.0922,367.0788,313.0703,283.0600,165.0175,121.0282	C_21_H_20_O_10_	[M + H]+
15	12.613	463.0883	Kaempferol 3-glucuronide	287.0556,113.0234	C_21_H_18_O_12_	[M + H]+
16	13.602	268.1328	Apomorphine	251.1064,237.0821,219.0803	C_17_H_17_NO_2_	[M + H]+
17	13.938	493.1335	Tricin 5-glucoside	331.0823,298.0466,316.0575,270.0516,197.0440	C_23_H_24_O_12_	[M + H]+
18	14.649	287.0551	Luteolin	219.0271,153.0185	C_15_H_10_O_6_	[M + H]+
19	17.079	271.0599	Baicalein	253.0487,225.0533,169.0128,123.0070	C_15_H_10_O_5_	[M + H]+
20	17.717	287.0909	Naringenin 5-methyl ether	269.0789,167.0333,121.0281	C_16_H_14_O_5_	[M + H]+
21	18.175	477.1014	6-*O*-Methylscutellarin	301.0705,286.0465	C_22_H_20_O_12_	[M + H]+
22	18.998	417.1188	Daidzin	255.0635	C_21_H_20_O_9_	[M + H]+
23	21.226	347.0777	5,7,3′,4′-Tetrahydroxy-6,8-dimethoxyflavone	317.0289,289.0343,182.9921,154.7773,135.0435	C_17_H_14_O_8_	[M + H]+
24	21.237	285.0740	Glycitein	270.0519,225.1122,167.0841	C_16_H_12_O_5_	[M + H]+
25	22.761	301.0700	Diosmetin	286.0463,183.0297,168.0048	C_16_H_12_O_6_	[M + H]+
26	22.973	523.1452	Centaurein	361.0908,331.0449,313.0338	C_24_H_26_O_13_	[M + H]+
27	23.538	178.1226	Metamfepramone	133.0648,105.0701	C_11_H_15_NO	[M + H]+
28	24.188	271.0597	Apigenin	153.0186,123.0078	C_15_H_10_O_5_	[M + H]+
29	25.595	269.0780	7-Hydroxy-3-(2-methoxyphenyl)-4H-chromen-4-one	253.0476,225.0527,197.0583,169.0635	C_16_H_12_O_4_	[M + H]+
30	25.916	285.0762	Wogonin	270.2516,252.0552,179.1482	C_16_H_12_O_5_	[M + H]+
31	29.958	345.0968	Eupatorin	315.0524,297.0419,197.0097,169.0146	C_18_H_16_O_7_	[M + H]+
32	31.544	267.1393	Honokiol	251.0919,226.0840,225.0763,211.0609,199.0603	C_18_H_18_O_2_	[M + H]+
33	32.394	267.1391	Magnolol	249.1129,225.0662	C_18_H_18_O_2_	[M + H]+
34	33.882	423.1081	6,8-Diprenylorobol	367.1194,311.0565,189.0906,147.0439	C_25_H_26_O_6_	[M + H]+
35	34.065	279.1598	2-(((2-Ethylhexyl)oxy)carbonyl)benzoic acid	223.0943,149.0230	C_16_H_22_O_4_	[M + H]+
36	34.905	421.1648	Pomiferin	365.0998,347.0897,311.0498,165.0181	C_25_H_24_O_6_	[M + H]+

### Untargeted identification results in MN

**Figure 3.**
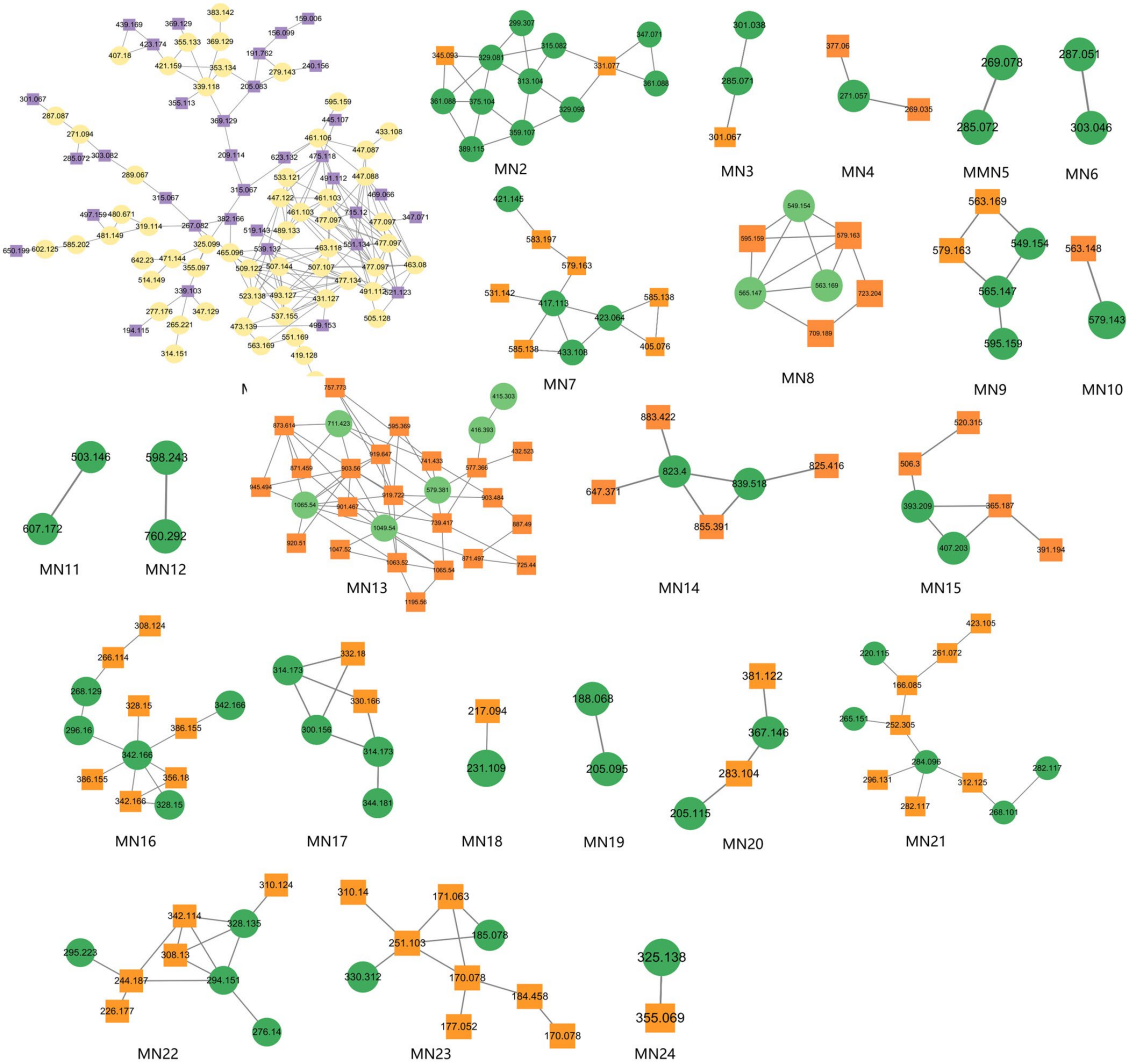
Twenty-four analyzable clusters obtained through molecular networking.

In this study, constituents with similar spectra were grouped into the same cluster. The relationships among the network nodes can be visualized using Cytoscape 3.9.2. Therefore, MN can be used to discover complex crude extracts of traditional Chinese medicine, effectively avoiding the drawbacks of low separation efficiency, high cost, and repetitive constituent isolation in traditional methods. In this study, a total of 24 clusters were analyzed, resulting in the identification of 180 constituents through MN analysis (Figure 3). The constituent′ s names, retention times, molecular formulas, precursor ions, and fragment ions are shown in Table 2.

**Table 2.** The constituents in water extract of dayuanyin decoction annotated by UPLC-QTOF-MS and molecular network.

Number	Formula	m/z	tR(min)	MSMS	Identification	Mode	Type	Cluster
37	C_30_H_32_O_12_	585.1966	22.850	249.0754,197.0812,151.0751,133.0639,105.0332	Benzoylpaeoniflorin	[M + H]+	Monoterpenes	MN1/MN11
38	C_17_H_18_O_6_	319.1173	8.869	197.0800,179.0692,151.0745,133.0636,105.0337	Paeoniflorgenin	[M + H]+	Monoterpenes	MN1
39	C_23_H_28_O_12_	497.1656	5.635	335.1104,197.0794,179.0578,151.0740,121.0274	Oxypaeoniflorin	[M + H]+	Monoterpenes	MN1
40	C_23_H_28_O_11_	481.1703	8.869	319.1173, 197.0810,179.0706,151.0756,123.0770, 105.0341	(1a*R*,2*S*,5*S*,5a*R*)-5*b*-[(benzoyloxy)methyl]tetrahydro-5-hydroxy-2-methyl-2,5-methano-1H-3,4-dioxacyclobuta[*cd*]pentalen-1a(2H)-yl	[M + H]+	Monoterpenes	MN1
41	C_23_H_28_O_11_	481.1709	8.869	319.1181,197.0760,179.0650,151.0709,123.0798, 105.0297	Albiflorin	[M + H]+	Monoterpenes	MN1/MN11
42	C_23_H_22_O_13_	507.1138	17.766	331.0820,316.0577,301.301.0345	3,4,5-Trihydroxy-6-[5-hydroxy-2-(4-hydroxy-3-methoxyphenyl)-3-methoxy-4-oxochromen-7-yl]oxyoxane-2-carboxylic acid	[M + H]+	Flavones	MN1
43	C_23_H_22_O_12_	491.1184	21.154	315.0885,300.0649,285.0415,257.0455	Irisolidone 7-*O*-glucuronide	[M + H]+	Flavones	MN1
44	C_24_H_24_O_12_	505.1436	20.308	301.0767,153.0876,121.0979,109.1012	Neocomplanoside	[M + H]+	Flavones	MN1
45	C_24_H_24_O_13_	521.1294	23.109	317.0468,169.0506,137.0220	Pedaliin 6′’-Acetate	[M + H]+	Flavones	MN1
46	C_27_H_30_O_13_	563.1762	16.574	269.0797,213.0887	Glycyroside	[M + H]+	Flavones	MN1
47	C_22_H_22_O_10_	447.1286	18.696	285.0770,270.0536	Isoprunetin-7-*O*-glucoside	[M + H]+	Flavones	MN1
48	C_23_H_24_O_11_	477.1398	21.071	315.0868,300.0624,282.0518,254.0563	Cirsimarin	[M + H]+	Flavones	MN1
49	C_21_H_20_O_11_	449.1070	15.241	287.0705	Astragalin	[M + H]+	Flavones	MN1
50	C_27_H_31_O_15_+	595.1658	14.670	433.1119,271.0588	Pelargonin	M+	Flavones	MN1
51	C_21_H_26_O_12_	471.1397	10.436	163.0389	[2,6-dihydroxy-5-[3,4,5-trihydroxy-6-(hydroxymethyl)oxan-2-yl]oxycyclohex-3-en-1-yl] (*E*)-3-(3,4-dihydroxyphenyl)prop-2-enoate	[M + H]+	Phenylpropanoids	MN1
52	C_21_H_22_O_5_	355.1538	30.164	299.0543,179.0314	1-[2,4-Dihydroxy-6-methoxy-3-(3-methyl-2-buten-1-yl)phenyl]-3-(4-hydroxyphenyl)-2-propen-1-one	[M + H]+	Phenylpropanoids	MN1
53	C_16_H_18_O_9_	355.1020	6.060	193.0503,163.0384,145.0274,135.0434,117.0329	Chlorogenic Acid	[M + H]+	Phenylpropanoids	MN1
54	C_20_H_20_O_5_	369.1335	32.940	313.0698,299.0537,179.0353	Desmethylxanthohumol	[M + H]+	Phenylpropanoids	MN1
55	C_16_H_18_O_8_	339.1073	15.670	191.0851,145.0285,117.0396	5-*p*-Coumaroylquinic acid	[M + H]+	Phenylpropanoids	MN1
56	C_16_H_20_O_7_	325.1287	11.899	191.0866,175.0741,119.0851	(1*R*,3*R*,4*S*,5*R*)-1,3,4-trihydroxy-5-[(*E*)-3-(4-hydroxyphenyl)prop-2-enoxy]cyclohexane-1-carboxylic acid	[M + H]+	Phenylpropanoids	MN1
57	C_29_H_36_O_15_	625.2120	14.436	479.1194,317.0665	Forsythoside A	[M + H]+	Phenylpropanoids	MN1
58	C_17_H_26_O_4_	277.176	26.243	177.0905,162.0668,145.0644,137.0593,117.0696	(5*R*)-5-Hydroxy-1-(4-hydroxy-3-methoxyphenyl)decan-3-one	[M + H-H2O]+	Others	MN1
59	C_23_H_24_O_13_	509.1296	12.853	347.0758	Syringetin-3-*O*-glucoside	[M + H]+	Flavones	MN1
60	C_22_H_22_O_9_	431.1331	16.929	269.0800,137.0235	Ononin	[M + H]+	Flavones	MN1
61	C_22_H_20_O_11_	461.1093	19.646	285.0750,270.0516	Oroxindin	[M + H]+	Flavones	MN1
62	C_16_H_14_O_5_	287.0915	20.675	183.0283,168.0047,140.0096	Dihydrooroxylin A	[M + H]+	Flavones	MN1
63	C_16_H_12_O_6_	301.0705	24.892	286.0469,258.0524,169.0123,141.0105	Sorbifolin	[M + H]+	Flavones	MN1
64	C_26_H_30_O_13_	551.1760	16.612	419.1335,259.0811,137.0227	Liquiritin apioside	[M + H]+	Flavones	MN1
65	C_25_H_26_O_5_	407.1852	33.911	351.1227,295.0604,283.0601,131.1394	Isolupalbigenin	[M + H]+	Flavones	MN1
66	C_25_H_26_O_7_	439.1752	33.394	421.3113, 383.1113, 109.0996	Millewanin G	[M + H]+	Flavones	MN1
67	C_25_H_26_O_6_	423.1805	33.298	367.1168,311.0551,283.0589,241.0493,165.0178	Mulberrin	[M + H]+	Flavones	MN1
68	C_21_H_22_O_9_	419.1337	11.707	257.0794,137.0228	Neoliquiritin	[M + H]+	Flavones	MN1
69	C_15_H_12_O_6_	289.0709	16.554	271.0613,247.0589,153.0186	(2*S*)-2′,5,6′,7-Tetrahydroxyflavanone	[M + H]+	Flavones	MN1
70	C_24_H_26_O_14_	539.1398	13.307	377.0860,362.0623,347.0391,137.0594	Limocitrol 3-β-D-glucoside	[M + H]+	Flavones	MN1
71	C_21_H_18_O_12_	463.0874	12.515	287.0557	Luteolin 7-glucuronide	[M + H]+	Flavones	MN1
72	C_22_H_20_O_13_	493.0983	21.543	317.1011	Isorhamnetin 3-glucuronide	[M + H]+	Flavones	MN1
73	C_21_H_18_O_11_	447.0927	17.054	271.0630	Baicalin	[M + H]+	Flavones	MN1
74	C_21_H_20_O_12_	465.1027	7.073	303.0483,165.0165,153.0167,149.0219,137.0221, 127.0375, 109.0279	Quercetin 3-Galactoside	[M + H]+	Flavones	MN1
75	C_23_H_22_O_11_	475.1237	24.376	285.0779,270.0541	Acacetin 7-*O*-D-methylglucuronate	[M + H]+	Flavones	MN1
76	C_25_H_24_O_13_	533.1288	20.187	285.0747,270.0517	6′’-*O*-Malonylglycitin	[M + H]+	Flavones	MN1
77	C_16_H_14_O_4_	271.0962	23.050	167.0329,152.0097,124.0148	Alpinetin	[M + H]+	Flavones	MN1
78	C_22_H_23_O_11_+	463.1239	17.775	301.0690,286.0456	Peonidin-3-glucoside	M+	Flavones	MN1
79	C_21_H_20_O_6_	369.1335	32.523	313.0705,295.0608,179.0345	2′,4′,5-Trihydroxy-7-methoxy-3-prenylflavone	[M + H]+	Flavones	MN1
80	C_20_H_18_O_5_	339.1227	30.164	283.0596,165.0171,137.0230,121.0281	Licoflavone C	[M + H]+	Flavones	MN1
81	C_21_H_20_O_6_	369.1330	28.310	313.0721,285.0729,271.0578,137.0234	Artocarpetin A	[M + H]+	Flavones	MN1
82	C_2_OH_18_O_6_	355.1178	29.827	299.0558,173.0599,121.0287	8-Prenylkaempferol	[M + H]+	Flavones	MN1
83	C_22_H_22_O_6_	383.1426	30.064	327.0884,299.0912,191.0700,179.0622,149.0231	Artocarpetin B	[M + H]+	Flavones	MN1
84	C_24_H_24_O_11_	489.1390	22.430	285.0757,270.0518,109.0281	6′’-*O*-Acetylglycitin	[M + H]+	Flavones	MN1
85	C_25_H_26_O_12_	519.1496	22.597	315.0840,300.0594,285.0364,163.0508,151.0352	Scutellarein 6,4′-dimethyl ether 7-(6′’-acetylglucoside)	[M + H]+	Flavones	MN1
86	C_22_H_20_O_12_	477.1028	19.996	301.0689,286.0455,153.1342	Chrysoeriol glucuronide	[M + H]+	Flavones	MN1
87	C_27_H_26_O_17_	623.1242	16.679	447.0909,271.0598	Clerodendrin	[M + H]+	Flavones	MN1
88	C_23_H_22_O_11_	475.1234	25.063	285.0779,270.0543	Wogonin 7-*O*-beta-D-glucuronide methyl ester	[M + H]+	Flavones	MN1
89	C_22_H_20_O_10_	445.1125	24.376	255.0650,240.0574	Chrysin 7-*O*-glucuronide methyl ester	[M + H]+	Flavones	MN1
90	C_15_H_10_O_7_	303.0498	10.915	257.0432,247.0591,229.0480,153.0711,149.0222	Morin	[M + H]+	Flavones	MN1
91	C_17_H_16_O_4_	285.0759	18.933	270.0527,242.0580,167.0344,152.0109,124.0160	5,7-Dimethoxyflavonone	[M + H]+	Flavones	MN1
92	C_17_H_14_O_8_	347.0761	11.086	301.0703,283.0593,265.0507,237.0340,137.0234,109.1015	Eupatoletin	[M + H]+	Flavones	MN1
93	C_12_H_14_O_4_	240.1237	26.372	208.1335,180.1382,109.0600	Methyl propyl phthalate	[M + NH4]+	Others	MN1
94	C_16_H_22_O_4_	279.1592	34.082	149.0231,121.0281	Dibutyl Phthalate	[M + H]+	Others	MN1
95	C_15_H_22_O_9_	347.1338	6.643	185.0797,125.0600	2-[(4-Hydroxy-3,5-dimethoxyphenyl)methoxy]-6-(hydroxymethyl)oxane-3,4,5-triol	[M + H]+	Others	MN1
96	C_12_H_12_O_3_	205.0858	34.077	163.0330, 149.0230,121.0278,	methyl 2-[(*E*)-but-2-enoyl]benzoate	[M + H]+	Others	MN1
97	C_11_H_10_O_3_	191.0704	6.093	149.1067,121.0755	Prop-2-enyl 2-formylbenzoate	[M + H]+	Others	MN1
98	C_18_H_16_O_8_	361.0917	22.138	331.0450,313.0341,285.0391,197.0086,169.0129	Isothymonin	[M + H]+	Flavones	MN2
99	C_17_H_14_O_6_	315.0869	28.927	285.0383,257.0434,182.9916,154.9968	7,8-Dihydroxy-5,6-dimethoxy-2-phenylchromen-4-one	[M + H]+	Flavones	MN2
100	C_18_H_16_O_8_	361.0919	23.696	331.0454,303.0504,182.9918	5,2′,6′-Trihydroxy-6,7,8-Trimethoxyflavone	[M + H]+	Flavones	MN2
101	C_18_H_16_O_7_	345.0965	28.464	330.0697,329.0652,312.0608,287.0530	Santin	[M + H]+	Flavones	MN2
102	C_20_H_20_O_8_	389.1231	25.926	359.0749,344.0519,313.0698,211.0238,183.0289	3′-Demethylnobiletin	[M + H]+	Flavones	MN2
103	C_17_H_14_O_7_	331.0817	24.442	301.0334,273.0377,182.9935,154.9979	Quercetin 3,4′-dimethyl ether	[M + H]+	Flavones	MN2
104	C_17_H_14_O_8_	347.0765	15.728	317.0304,289.0349,182.9930,154.9977	5,6,7,3′-Tetrahydroxy-3,4′-dimethoxyflavone	[M + H]+	Flavones	MN2
105	C_18_H_16_O_6_	329.1023	23.846	299.0538,271.0590,153.0179,135.0043,125.0225,121.0276	6-Hydroxy-5,7,4′-Trimethoxyflavone	[M + H]+	Flavones	MN2
106	C_18_H_16_O_8_	361.0921	23.814	331.0441,303.0503,182.9918,121.0644	Jaceidin	[M + H]+	Flavones	MN2
107	C_19_H_18_O_8_	375.1078	29.227	345.0603,327.0494,197.0081,169.0131	Skullcapflavone II	[M + H]+	Flavones	MN2
108	C_15_H_10_O_7_	303.0498	10.915	257.0432,229.0480,153.0189,137.0238	Quercetin	[M + H]+	Flavones	MN2
109	C_18_H_16_O_5_	313.1070	27.085	298.0830,283.0601,269.0803,255.0650	2′,5,6-Trimethoxyflavone	[M + H]+	Flavones	MN2
110	C_16_H_12_O_5_	285.0759	18.933	270.0522,242.0580,167.0341	Oroxylin A	[M + H]+	Flavones	MN3
111	C_16_H_12_O_6_	301.0708	23.538	286.0462,258.0499,183.9997	Hispidulin	[M + H]+	Flavones	MN3
112	C_16_H_12_O_6_	301.0709	22.755	286.0466,258.0515,140.0102,168.0050	Tectorigenin	[M + H]+	Flavones	MN3
113	C_16_H_12_O_4_	269.0809	24.680	251.0696,223.0739,167.0851,152.0615,103.0537	5-Hydroxy-6-Methoxyflavone	[M + H]+	Flavones	MN4
114	C_16_H_12_O_4_	269.0808	25.595	254.0565,237.0536,213.0905,197.0590	Formononetin	[M + H]+	Flavones	MN5
115	C_16_H_12_O_5_	285.0759	18.933	270.0527,253.0496,225.0547	Calycosin	[M + H]+	Flavones	MN5
116	C_15_H_10_O_6_	287.0553	14.632	153.0811,121.023	Kaempferol	[M + H]+	Flavones	MN6
117	C_15_H_10_O_7_	303.0496	10.915	257.0455,229.0483,179.0330,153.0174,137.0226	Viscidulin I	[M + H]+	Flavones	MN6
118	C_20_H_20_O_9_	405.1187	11.861	387.0689,369.0589,351.0483,339.0496,327.0483,273.0380	3-β-D-Glucopyranosyl-1-hydroxy-7-methoxy-9H-xanthen-9-one	[M + H]+	Flavones	MN7
119	C_25_H_28_O_16_	585.1458	6.318	423.0981,369.0574,339.0460,327.0473,303.0476,273.0367	2-((2*S*,3*R*,4*R*,5*S*,6*R*)-3,4-Dihydroxy-6-(hydroxymethyl)-5-(((2*R*,3*R*,4*S*,5*S*,6*R*)-3,4,5-trihydroxy-6-(hydroxymethyl)tetrahydro-2H-pyran-2-yl)oxy)tetrahydro-2H-pyran-2-yl)-1,3,6,7-tetrahydroxy-9H-xanthen-9-one	[M + H]+	Flavones	MN7
120	C_20_H_20_O_10_	421.1142	13.786	367.1183,337.1092,325.1078,301.1077,271.0968	1,3,6,7-Tetrahydroxy-2-(2,3,4-trihydroxy-5-(hydroxymethyl)cyclohexyl)-9H-xanthen-9-one	[M + H]+	Flavones	MN7
121	C_21_H_20_O_9_	417.1189	14.745	399.1075,297.0760,279.0644,267.0649,167.0696	Puerarin	[M + H]+	Flavones	MN7
122	C_27_H_30_O_14_	579.1704	11.328	441.1163,363.0843,321.0738,309.0739,279.0634	5,7-Dihydroxy-2-phenyl-6,8-bis[3,4,5-trihydroxy-6-(hydroxymethyl)oxan-2-yl]chromen-4-one	[M + H]+	Flavones	MN7
123	C_27_H_30_O_13_	563.1710	14.174	441.1173,431.1099,387.0866,363.0852,339.0852,321.0747,309.0748,279.0644	6,8-bis(6-Deoxy-α-L-mannopyranosyl)-5,7-dihydroxy-2-(4-hydroxyphenyl)-4H-1-benzopyran-4-one	[M + H]+	Flavones	MN8
124	C_27_H_30_O_15_	595.1659	9.223	577.1554,457.1129,337.0708	Vicenin 2	[M + H]+	Flavones	MN8/MN9
125	C_26_H_28_O_13_	549.1610	12.524	459.1041,429.0955,411.1079,393.0969,375.0860,363.0856,339.0862,309.0751	Chrysin 6-C-glucoside 8-C-arabinoside	[M + H]+	Flavones	MN8/MN9
126	C_26_H_28_O_14_	565.1559	10.769	547.1447,529.1327,511.1233,475.1028,445.1119,427.1013,355.0794,325.0700,	Isoschaftoside	[M + H]+	Flavones	MN8/MN9
127	C_27_H_30_O_14_	579.1704	11.328	441.1163,363.0843,321.0738,309.0739,279.0634	Chrysin 6,8-di-C-glucoside	[M + H]+	Flavones	MN8/MN9
128	C_27_H_30_O_13_	563.1760	16.058	441.1173,363.0852,321.0747,309.0748	5,7-Dihydroxy-2-phenyl-6-(3,4,5-trihydroxy-6-(hydroxymethyl)tetrahydro-2H-pyran-2-yl)-8-(3,4,5-trihydroxy-6-methyltetrahydro-2H-pyran-2-yl)-4H-chromen-4-one	[M + H]+	Flavones	MN9
129	C_30_H_26_O_11_	563.1487	6.881	411.1052,291.0850,255.0642,107.0492	Gambiriin C	[M + H]+	Flavones	MN10
130	C_34_H_46_O_18_	760.3006	11.494	582.4137,420.1126,282.2164,250.2865,222.0876	Liriodendrin	[M + NH4]+	Phenylpropanoids	MN12
131	C_28_H_36_O_13_	598.2498	14.716	420.1135,282.2158,250.2671,222.0945	Acanthoside B	[M + NH4]+	Phenylpropanoids	MN12
132	C_51_H_86_O_24_	1065.5534	18.300	903.4923,741.4403,579.3877,417.3354,273.2208,255.2109	Tomatoside A	M + H-H2O	Steroids	MN13
133	C_51_H_84_O_24_	1063.5247	19.892	901.4746,739.4233,577.3715,415.3193,397.3061,271.2027,253.1924	(3β,22α,25S)-26-(β-D-Glucopyranosyloxy)-22-hydroxyfurost-5-en-3-yl *O*-β-D-glucopyranosyl-(1→2)-*O*-β-D-glucopyranosyl-(1→4)-β-D-glucopyranoside	M + H-H2O	Steroids	MN13
134	C_45_H_76_O_19_	903.5601	20.692	741.4395,579.3873,417.3357,273.2204,255.2097	Chamaedroside E	M + H-H2O	Steroids	MN13
135	C_47_H_78_O_20_	945.5043	23.217	783.4537,621.4004,417.3355,399.3256,273.2217,255.2101	(4,5-Dihydroxy-6-((10-hydroxy-6a,8a,9-trimethyl-10-(3-methyl-4-((3,4,5-trihydroxy-6-(hydroxymethyl)tetrahydro-2H-pyran-2-yl)oxy)butyl)octadecahydro-1H-naphtho[2′,1′:4,5]indeno[2,1-b]furan-4-yl)oxy)-3-((3,4,5-trihydroxy-6-(hydroxymethyl)tetrahydro-2H-pyran-2-yl)oxy)tetrahydro-2H-pyran-2-yl)methyl acetate	M + H-H2O	Steroids	MN13
136	C_51_H_86_O_23_	1049.5544	19.108	887.5023,741.4426,579.3908,415.3377,273.2220,255.2114	(3β,5β,22α,25S)-26-(β-D-Glucopyranosyloxy)-22-hydroxyfurostan-3-yl *O*-6-deoxy-α-L-mannopyranosyl-(1→2)-*O*-[β-D-glucopyranosyl-(1→4)]-β-D-glucopyranoside	M + H-H2O	Steroids	MN13
137	C_51_H_84_O_23_	1065.5475	19.187	903.4925,741.4418,579.3895,417.3365,399.3253,273.2207,255.2103	(25*R*)-3β-[(2-*O*,4-*O*,6-*O*-Tri-β-D-glucopyranosyl-β-D-glucopyranosyl)oxy]-5β-spirostane	[M + H]+	Steroids	MN13
138	C_51_H_82_O_22_	1047.5372	18.787	885.4809,739.4217,577.3711,415.3201,271.2205,253.2100	Graecunin E	[M + H]+	Steroids	MN13
139	C_38_H_62_O_12_	711.4321	27.280	549.3777,417.3398,273.2215,255.2108	(3β,5β,25*S*)-Spirostan-3-yl 6-*O*-β-D-xylopyranosyl-β-D-glucopyranoside	[M + H]+	Steroids	MN13
140	C_44_H_72_O_17_	873.4853	21.638	711.4327,567.3162,417.3373,399.3261, 273.2218,255.2214	Schidegera saponin D1	[M + H]+	Steroids	MN13
141	C_44_H_70_O_17_	871.4684	21.300	709.4149,415.3208,397.3084,271.2209,253.2103	Capsicoside C3	[M + H]+	Steroids	MN13
142	C_45_H_72_O_18_	901.4791	19.041	739.4264,577.3737,415.3215,271.2214,253.2109	Funkioside D	[M + H]+	Steroids	MN13
143	C_33_H_54_O_8_	579.3899	24.805	417.3381,273.2219,255.2109	Asparagoside A	[M + H]+	Steroids	MN13
144	C_33_H_54_O_9_	595.3842	22.780	577.3714,433.3301,415.3193(母核脱水后),273.2202,255.2096	Gitogenin 3-galactoside	[M + H]+	Steroids	MN13
145	C_33_H_52_O_8_	577.3739	20.254	415.3184,271.2045,253.1935	Disogluside	[M + H]+	Steroids	MN13
146	C_39_H_62_O_13_	739.4262	20.150	577.3708,433.2558,271.2047,253.1936	Diosgenin gentiobioside	[M + H]+	Steroids	MN13
147	C_39_H_64_O_12_	725.4475	29.593	417.2521,273.2210,255.2104	Smilanippin A	[M + H]+	Steroids	MN13
148	C_45_H_74_O_16_	871.5057	29.043	417.2725,273.2205,255.2101	Indioside I	[M + H]+	Steroids	MN13
149	C_45_H_74_O_17_	887.4996	27.401	433.3188,271.2044,253.1940	26-Deglucoprotodioscin	[M + H]+	Steroids	MN13
150	C_45_H_74_O_18_	903.4948	26.147	741.4402,415.3360, 271.2059,253.1952	Protobioside	[M + H]+	Steroids	MN13
151	C_45_H_74_O_19_	919.4894	16.424	757.4364,595.3835,433.3312,415.3199,289.2160,271.2050	[(25*S*)-2α-Hydroxy-5α-spirostan-3β-yl]4-*O*-(2-*O*-β-D-galactopyranosyl-β-D-glucopyranosyl)-β-D-galactopyranoside	[M + H]+	Steroids	MN13
152	C_45_H_74_O_19_	919.4893	17.829	757.4341,595.3830,433.3312,415.3200,271.2047,253.2939	Melongoside O	[M + H]+	Steroids	MN13
153	C_39_H_64_O_14_	757.4363	26.674	595.3813,433.3287,415.3182,271.2042,253.1939	Lilioglycoside	[M + H]+	Steroids	MN13
154	C_27_H_44_O_3_	417.3368	30.304	273.2212,255.2104	3-Episarsasapogenin	[M + H]+	Steroids	MN13
155	C_27_H_45_NO_2_	416.3527	22.280	398.3070,273.2202,255.2094	Soladulcidine	[M + H]+	Steroids	MN13
156	C_39_H_66_O_14_	741.4334	28.122	417.3388,399.3287,381.3184,271.3082,253.1974	26-*O*-Des-β-D-glucopyranosylbethoside B	M + H-H2O	Steroids	MN13
157	C_42_H_62_O_16_	823.4116	28.213	647.3780,471.3444,453.3373,407.3283,217.1571,195.1704,149.1319	Liquorice	[M + H]+	Triterpenes	MN14
158	C_44_H_66_O_18_	883.4278	26.593	707.3961,531.3580,471.3461,453.3355,435.3242,353.0714	22β-Acetoxylicoricesaponin J2	[M + H]+	Triterpenes	MN14
159	C_42_H_62_O_17_	839.4049	26.776	663.3728,487.3405,469.3302	Licoricesaponin G2	[M + H]+	Triterpenes	MN14
160	C_42_H_64_O_16_	825.3906	26.939	663.3743,487.3429,469.3321	Quillaic acid 3-[galactosyl-(1->2)-glucuronide]	[M + H]+	Triterpenes	MN14
161	C_36_H_54_O_10_	647.3797	28.213	471.3446,453.3365,435.3249,407.3305,217.1580,149.1320	Glycyrrhetic Acid 3-*O*-Glucuronide	[M + H]+	Triterpenes	MN14
162	C_42_H_62_O_18_	855.4009	22.450	679.3689,503.3376,485.3269,467.3147	22-Hydroxy-licorice-saponin G2	[M + H]+	Triterpenes	MN14
163	C_24_H_40_O_4_	393.2997	4.964	375.2120,357.2012,339.1904,309.1796	Deoxycholatic acid	[M + H]+	Steroids	MN15
164	C_25_H_42_O_4_	407.2385	8.115	389.2243,371.2140,353.2033,323.1931	Methyl deoxycholate	[M + H]+	Steroids	MN15
165	C_22_H_36_O_4_	365.2690	1.777	347.1805,329.1703,311.1590,281.1480	Dinorhyodeoxycholic Acid	[M + H]+	Steroids	MN15
166	C_24_H_38_O_4_	391.2844	4.689	373.1986,355.1872,307.1671	12-Ketolithocholic acid	[M + H]+	Steroids	MN15
167	C_17_H_17_NO_2_	268.1332	13.049	251.1053,219.0791,191.0843	Asimilobine	[M + H]+	Alkaloids	MN16
168	C_17_H_15_NO_2_	266.1178	17.958	249.0901,219.0788,191.0844	1-Hydroxy-5,6,6a,7-tetrahydro-4H-dibenzo[*de,g*]quinoline-2-carbaldehyde	[M + H]+	Alkaloids	MN16
169	C_17_H_15_NO_2_	266.1178	17.958	249.0901,219.0788,191.0844	(*R*)-2-Hydroxy-5,6,6a,7-tetrahydro-4H-dibenzo[*de,g*]quinoline-1-carbaldehyde	[M + H]+	Alkaloids	MN16
170	C_20_H_24_NO_4_+	342.1709	9.594	297.1112,282.0823,265.0803,237.0901	Magnoflorine	M+	Alkaloids	MN16
171	C_21_H_24_NO_6_+	386.1596	8.173	341.0974,326.0740,309.0714,296.0990,281.0763,237.0866,205.0637	3-Carboxy-1,11-dihydroxy-2,10-dimethoxy-6,6-dimethyl-5,6,6a,7-tetrahydro-4H-dibenzo[*de,g*]quinolin-6-ium	M+	Alkaloids	MN16
172	C_18_H_19_NO_3_	298.1435	17.229	251.1042,219.0781,191.0843	*N*-Methylasimilobine *N*-oxide	[M + H]+	Alkaloids	MN16
173	C_21_H_26_NO_4_+	356.1850	16.168	311.1284,296.1040,279.1094,264.0847	Menispermine	M+	Alkaloids	MN16
174	C_19_H_22_NO_4_+	328.1540	7.385	283.0973,251.0709,223.0747, 205.0629,177.0679	1,9,10-Trihydroxy-2-methoxy-6,6-dimethyl-5,6,6a,7-tetrahydro-4H-dibenzo[*de,g*]quinoline-6-ium	M+	Alkaloids	MN16
175	C_20_H_24_NO_4_+	342.1708	11.690	297.1117,265.0856,237.0899	Laurifoline	M+	Alkaloids	MN16
176	C_19_H_21_NO_4_	328.1544	11.636	297.1111,265.0849,237.0898,205.0635,177.0681	Boldine	[M + H]+	Alkaloids	MN16
177	C_18_H_21_NO_3_	300.1596	8.040	269.1155,237.0893,175.0741,107.0489	(*S*)-*N*-Methylcoclaurine	[M + H]+	Alkaloids	MN17
178	C_19_H_23_NO_5_	346.1641	8.544	299.1275,192.1006,175.0745,137.0591	Reticuline N-oxide	[M + H]+	Alkaloids	MN17
179	C_19_H_24_NO_3_+	314.1754	6.289	269.1169,237.0900,209.0949,175.0749,143.0487,107.0491	(-)-Lotusine	M+	Alkaloids	MN17
180	C_19_H_25_NO_4_	332.1854	10.198	314.1748,269.1166,237.0901,209.0942,175.0753,107.0488	Isoquinolinium, 1,2,3,4-tetrahydro-6-hydroxy-1-(*p*-hydroxybenzyl)-7-methoxy-2,2-dimethyl-, hydroxide	M+	Alkaloids	MN17
181	C_19_H_24_NO_3_+	314.1750	6.402	269.1172,237.0902,209.0952,175.0753,107.0494	1-[(4-Hydroxyphenyl)methyl]-6-methoxy-2,2-dimethyl-3,4-dihydro-1H-isoquinolin-2-ium-7-ol	M+	Alkaloids	MN17
182	C_19_H_24_NO_4_+	330.1703	5.064	285.1108,269.1158,237.0879,209.0954,175.0726,107.0482	1-(3,4-Dihydroxybenzyl)-2,2-dimethyl-6-methoxy-7-hydroxy-1,2,3,4-tetrahydroisoquinoline-2-ium	M+	Alkaloids	MN17
183	C_13_H_14_N_2_O_2_	231.1123	6.548	214.0862,188.0704,158.0964, 130.0654	Tetrahydroharman-3-carboxylic acid	[M + H]+	Amino acids	MN18
184	C_12_H_12_N_2_O_2_	217.0970	5.952	200.1271,171.0905,144.0802,130.0654	2,3,4,9-Tetrahydro-1H-beta-carboline-3-carboxylic acid	[M + H]+	Amino acids	MN18
185	C_11_H_9_NO_2_	188.0706	3.943	170.0585,146.0595,118.0644	3-Indoleacrylic acid	[M + H]+	Amino acids	MN19
186	C_11_H_12_N_2_O_2_	205.0977	3.939	188.0704,170.0597,146.0604,118.0655	Tryptophan	[M + H]+	Amino acids	MN19/MN20
187	C_17_H_22_N_2_O_7_	367.1510	3.785	349.1410,332.1139,303.1341,188.0709,170.0598,146.0600,118.0651	1-beta-D-Glucopyranosyl-L-tryptophan	[M + H]+	Amino acids	MN20
188	C_18_H_14_N_2_O_7_	381.1650	0.848	219.0258,201.0144,173.0307,118.0877	1-[[1-Carboxy-2-(1H-indol-3-yl)ethyl]amino]-1-deoxyfructose, 9CI	[M + H]+	Amino acids	MN20
189	C_11_H_12_N_3_O_4_S-	283.0624	7.769	188.0696,170.0587,146.0593,118.0645	3-[(2*S*)-2-Amino-3-oxo-3-(sulfinatoamino)oxypropyl]-1H-indole	[M + H]+	Amino acids	MN20
190	C_10_H_13_N_5_O_5_	284.0971	1.377	152.0564,135.0295,124.0388,110.0346	Guanosine	[M + H]+	Adenosines	MN21
191	C_12_H_17_N_5_O_5_	312.1302	3.202	180.0883,135.0310,110.0355	*N*2,*N*2-Dimethylguanosine	[M + H]+	Adenosines	MN21
192	C_11_H_15_N_5_O_4_	282.1192	2.010	136.0615,119.0341,101.0952	2′-*O*-Methyladenosine	[M + H]+	Adenosines	MN21
193	C_10_H_13_N_5_O_4_	268.1015	1.394	136.0620,119.0349	Adenosine	[M + H]+	Adenosines	MN21
194	C_12_H_17_N_5_O_4_	296.0350	4.460	248.1253,164.0898,136.0724, 119.0781	*N*6-Ethyladenosine	[M + H]+	Adenosines	MN21
195	C_11_H_15_N_5_O_4_	282.1196	2.410	151.0607, 136.0607,119.0341	*N*6-Methyladenosine	[M + H]+	Adenosines	MN21
196	C_14_H_20_N_2_O_3_	265.1547	5.856	248.1274, 219.1471,177.0545,120.0798,103.0541	Phe-Val	[M + H]+	Amino acids	MN21
197	C_12_H_17_N_3_O_3_	252.0866	9.452	206.0798,120.0767,103.0544	3-Amino-2-(2-amino-3-phenylpropanamido)propanoic acid	[M + H]+	Amino acids	MN21
198	C_18_H_19_NO_4_	314.1385	17.883	177.0554,145.0286,121.0650,117.0334,103.0542	*N*-*trans*-Feruloyltyramine	[M + H]+	Amino acids	MN21
199	C_14_H_20_N_2_O_3_	265.1539	5.856	248.1267,177.0543,145.0280,117.0325	Feruloylputrescine	[M + H]+	Amino acids	MN21
200	C_12_H_23_NO_7_	294.1549	1.356	276.1458,258.1352,230.1402,212.1291	3-Methyl-2-[[2,3,4-trihydroxy-5-(hydroxymethyl)oxolan-2-yl]methylamino]pentanoic acid	[M + H]+	Amino acids	MN22
201	C_12_H_23_NO_7_	276.1436	1.352	276.1458,258.1352,230.1402,212.1291	3-Methyl-2-[[2,3,4-trihydroxy-5-(hydroxymethyl)oxolan-2-yl]methylamino]pentanoic acid	[M + H-H2O]+	Amino acids	MN22
202	C_15_H_21_NO_7_	328.1390	2.156	310.1287,292.1182,282.1328, 264.1232,178.0863,166.0863,120.0808	*N*-Fructosyl phenylalanine	[M + H]+	Amino acids	MN22
203	C_11_H_19_NO_9_	310.1242	2.168	292.1164,264.1210,166.0844,120.0796	L-Glutamic acid, *N*-(1-deoxy-α-D-fructofuranos-1-yl)-	[M + H]+	Amino acids	MN22
204	C_11_H_19_NO_9_	310.1242	2.168	292.1164,264.1210,166.0844,120.0796	L-Glutamic acid, *N*-(1-deoxy-β-D-fructofuranos-1-yl)-	[M + H]+	Amino acids	MN22
205	C_16_H_23_NO_7_	342.1140	2.410	324.1068,306.0963,278.1012,166.0856,120.0801	L-Phenylalanine, *N*-(1-deoxy-β-D-fructopyranos-1-yl)-, methyl ester	[M + H]+	Amino acids	MN22
206	C_18_H_30_O_3_	295.2267	25.343	277.2160,151.1112,107.0850	9-Oxooctadeca-10,12-dienoic acid	[M + H]+	Others	MN22
207	C_13_H_22_O_3_	244.1901	24.222	226.2157,208.1700,180.1752,163.1435,121.1016,107.0862	4-Hydroxy-5,7-tridecadienoic acid	[M + NH4]+	Others	MN22
208	C_13_H_20_O_2_	226.1794	24.235	208.1667,180.1743,163.1456, 121.1014,107.0855	7,9,11-Tridecatrienoic acid	[M + NH4]+	Others	MN22
209	C_9_H_12_O_4_	185.0807	7.585	170.0565,153.0546,139.0392,125.0596,111.0438	2,3,5-Trimethoxyphenol	[M + H]+	Others	MN23
210	C_8_H_10_O_4_	171.0652	2.864	139.0385,111.0439	2,6-Dimethoxybenzene-1,4-Diol	[M + H]+	Others	MN23
211	C_8_H_9_O_4_-	170.0572	7.064	138.0528,110.0579	(1-Hydroxy-2,6-dimethoxy-4-oxo-2,5-cyclohexadienyl)radical	[M + H]+	Others	MN23
212	C_9_H_11_O_4_-	184.0731	8.794	152.0702,110.0606	3,4,5-Trimethoxyphenolate	[M + H]+	Others	MN23
213	C_8_H_9_O_4_-	170.0784	4.393	152.0677,110.0577	1,4-Benzenediol, 2,6-dimethoxy-, ion(1-)	[M + H]+	Others	MN23
214	C_17_H_14_O_7_	331.0809	24.442	316.0578,301.0340,273.2573,182.9917,154.9964	Cirsiliol	[M + H]+	Flavones	MN23
215	C_20_H_20_O_4_	325.1433	31.464	189.0908,149.0598,123.0439	4-(8-Prop-1-en-2-yl-3,4,8,9-tetrahydro-2H-furo[2,3-*h*]chromen-3-yl)benzene-1,3-diol	[M + H]+	Others	MN24
216	C_20_H_18_O_6_	357.1169	29.827	299.0558,257.0451,191.1051,153.0534,123.0266	Uncinanone B	[M + H]+	Others	MN24

### Unannotated analysis points of flavonoid constituents in MN


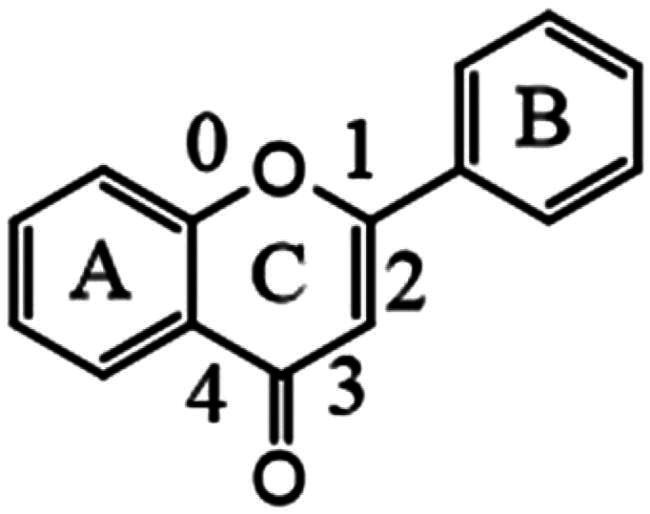


Flavonoid aglycone

**Figure 4.**
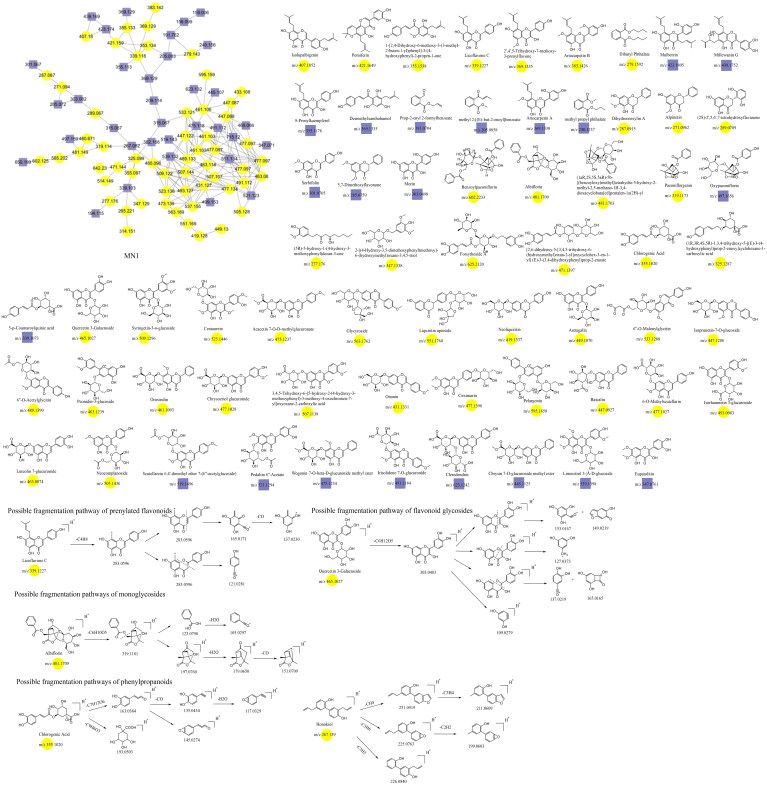
Structure of the MN1 constituents and the possible fragmentation pathway of some representative constituents.

**Figure 5.**
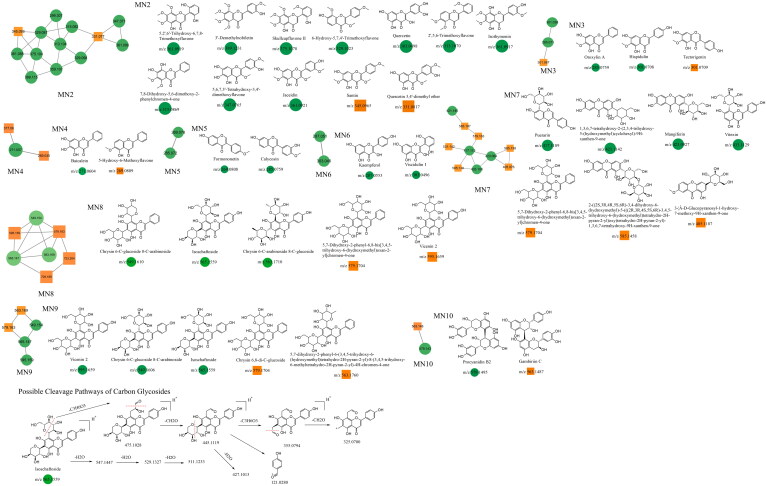
Structure of the MN2–MN10 constituents and the possible fragmentation pathway of a representative constituent.

The fragmentation of flavonoid constituents mainly involves the cleavage of glycosidic bonds and rings in the MS/MS spectra. In this study, we selected quercetin 3-galactoside and licoflavone C as representative constituents to investigate the MS/MS fragmentation patterns of flavonoids. Quercetin 3-galactoside (C_21_H_20_O_12_) easily forms a quasi-molecular ion [M + H]^+^ at *m/z* 465.1027 in the positive ion mode. The precursor ion loses one glucose molecule to generate a fragment ion at *m/z* 303.0483 ([M + H–C_6_H_10_O_5_]^+^). This fragment ion can undergo various types of fragmentation. One fragmentation pathway involves breaking the [0,4] bonds within the C ring, resulting in the formation of a fragment ion at *m/z* 109.0729 ([0,4A]^+^). Another possible fragmentation pathway is the cleavage of the chemical bond at the [1,3] positions, leading to the formation of fragment ions at *m/z* 153.0167 ([1,3A]^+^) and 149.0167 ([1,3B]^+^). Additionally, the fragment ion at *m/z* 303.0483 can undergo cleavage of the [1,4] chemical bond, generating a fragment ion at *m/z* 127.0373 (C_6_H_6_O_3_, [1,4A]^+^). Lastly, the fragment ion at *m/z* 303.0483 can also undergo cleavage of the [0,2] chemical bond, resulting in the formation of fragment ions at *m/z* 137.0219 and 165.0165 ([M + H–C_6_H_10_O_5_–C_7_H_6_O_3_]^+^). For licoflavone C, in the positive ion mode, the extracted ion chromatogram (EIC) of *m/z* 339.1227 (C_20_H_18_O_5_, [M + H]^+^) predominantly undergoes the loss of a tert-butyl group from the A ring to generate a fragment ion at *m/z* 283.0596 ([M + H–C_4_H_8_]^+^). This fragment ion can further undergo fragmentation at either the [1,3] bond or the [0,2] bond to produce the corresponding fragments. The former fragmentation results in a fragment ion at *m/z* 165.0171 ([M + H–C_8_H_6_O]^+^), whereas the latter generates a fragment ion at *m/z* 121.0281 (C_7_H_5_O_2_, [M + H]^+^). Additionally, the fragment ion at *m/z* 165.0171 can further lose a carbon monoxide (CO) molecule to yield a fragment ion at *m/z* 137.0230 ([M + H-C_8_H_6_O–CO]^+^). In addition to glycosides and oxygenated glycosides, the flavonoid constituents in Dayuanyin water decoction also included carbonated glycosides. Carbonated glycoside fragmentation analysis was conducted using isoschaftoside as an example. In the positive ion mode, the constituents initially form a quasi-molecular ion at *m/z* 565.1559 ([M + H]^+^). Subsequently, the sugar moiety attached to the flavonoid aglycone undergoes fragmentation. Following the consecutive loss of C_3_H_6_O_3_ and CH_2_O groups, characteristic fragment ions at *m/z* 445.1119 ([M + H–C_3_H_6_O_3_–CH_2_O]^+^) and 325.0700 ([M + H–2C_3_H_6_O_3_–2CH_2_O]^+^) are generated. The MS/MS spectrum is displayed above. A new constituent, 5,7-dihydroxy-2-phenyl-6-(3,4,5-trihydroxy-6-(hydroxymethyl)tetrahydro-2H-pyran-2-yl)-8-(3,4,5-trihydroxy-6-methyltetrahydro-2H-pyran-2-yl)-4H-chromen-4-one, might appear in carbonated glycosides. A comparison of this constituent to the hypothesized chrysin 6,8-di-C-glucoside showed that they both had the same characteristic fragment ions at *m/z* 441, 363, 321, 309, indicating that their structures were essentially similar. Additionally, as the molecular ions of the two constituents differed by 16 Da, it can be inferred that an oxygen (O) atom was missing from the OH group of the glucose moiety. Therefore, it is likely that constituent is 5,7-dihydroxy-2-phenyl-6-(3,4,5-trihydroxy-6-(hydroxymethyl)tetrahydro-2H-pyran-2-yl)-8-(3,4,5-trihydroxy-6-methyltetrahydro-2H-pyran-2-yl)-4H-chromen-4-one mentioned in the previous text. Among the studied constituent clusters, the flavonoid constituents mainly originated from MN1 to MN10. The structural formulas of flavonoid constituents in Dayuanyin water decoction are shown in Figures 4 and 5, and the mass spectrometry information is provided in Table 2.

### Unannotated analysis points of terpenoid constituents in MN

**Figure 6.**
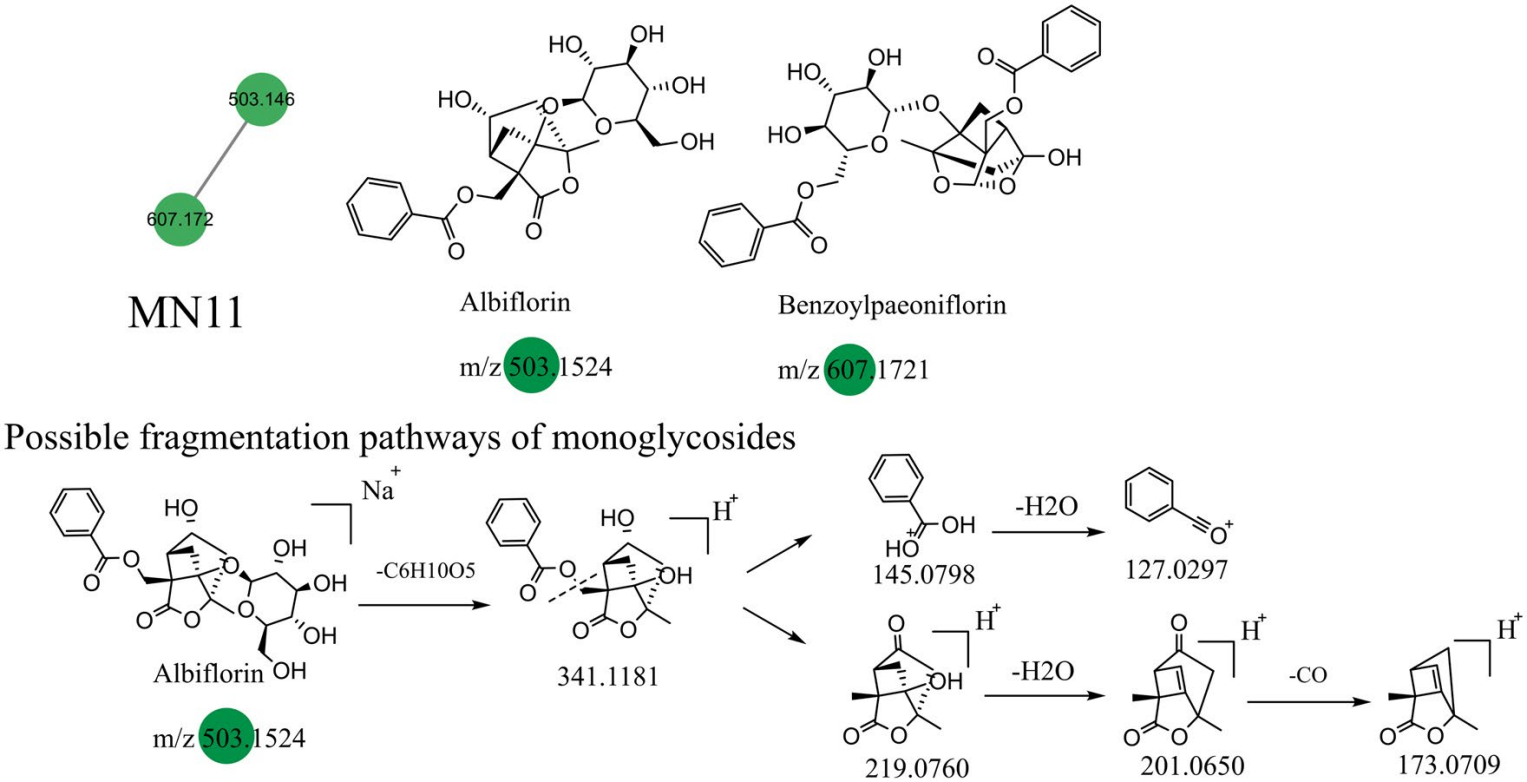
Structure of the MN11 constituents and the possible fragmentation pathway of a representative constituent.

The terpenoid constituents in Dayuanyin water decoction mainly included monoterpenes and sesquiterpenes, primarily derived from *Paeonia lactiflora*. Taking albiflorin (C_23_H_28_O_11_) as an example, its quasi-molecular ion peak in the positive ion mode is observed at *m/z* 481.1709. First, the molecular ion peak loses one glucose molecule, resulting in a fragment ion at *m/z* 319.1181. Then, the ester bond and the adjacent C-C bond of benzoic acid break, giving rise to two fragment ions: one at *m/z* 123.0798 (C_7_H_6_O_2_, [M + H–C_10_H_12_O_4_]^+^), representing the fragment of benzoic acid, and another at *m/z* 197.0760 (C_10_H_12_O_4_, [M + H-C_6_H_10_O_5_–C_7_H_6_O_2_]^+^). The former further loses one water molecule, generating a fragment ion at *m/z* 105.0297, while the latter sequentially loses one water molecule and CO, resulting in fragment ions at *m/z* 179.0650 and *m/z* 151.0709. The relevant fragmentation pathways are shown in Figure 6. The constituent oxypaeoniflorin, which lacks annotated markers, exhibits secondary fragment ions similar to those of albiflorin, including *m/z* 197/179/151. Additionally, the ions *m/z* 335 and *m/z* 121 are both 16 Da higher than the secondary fragment ions *m/z* 319 and *m/z* 105 present in albiflorin, suggesting the presence of a hydroxyl group on the benzene ring. The hypothesized molecular formula was matched with constituents in Scifinder, ultimately confirming that the annotated marker corresponded to oxypaeoniflorin. This class of constituents exhibits primary fragment ions, including *m/z* 197/179/151/105, such as benzoylpaeoniflorin, (1 *R*,2*S*,5*S*,5*R*)-5*b*-[(benzyloxy)-methyl]tetrahydro-5-hydroxy-2-methyl-2,5-methano-1H-3,4-dioxacyclobuta[*cd*]pentalen-1a(2H)-yl, oxypaeoniflorin, and paeoniflorgenin, which all contain these fragments. Therefore, it can be presumed that these constituents belong to the same class. The monoterpenoid constituents in this study primarily originated from MN1 and MN11. The relevant constituent information is shown in Table 2, and their structural formulas are shown in Figure 6.

### Unannotated analysis points of phenylpropane constituents in MN

**Figure 7.**
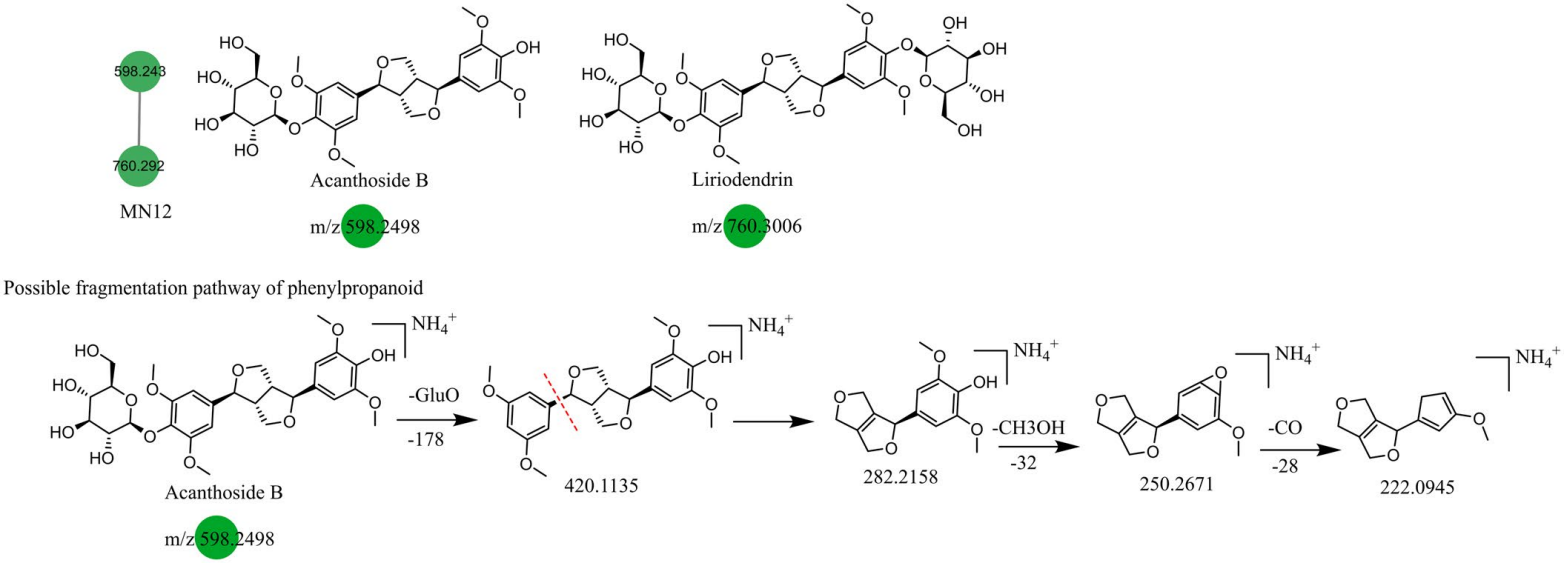
Structure of the MN12 constituents and the possible fragmentation pathway of a representative constituent.

The main phenylpropane constituents identified in the Dayuanyin decoction were phenylpropanol, phenylpropanoic acid, and lignan constituents derived from *Magnoliae Officinalis.* The lignan constituents tended to lose functional groups such as allyl, caffeoyl, and CO during the fragmentation process. The example used for fragmentation was honokiol. In positive ion mode detection, the quasi-molecular ion peak formed by honokiol is *m/z* 267.1390 ([M + H]^+^). After losing one molecule of allyl, the fragment ion *m/z* 225.0763 is generated ([M + H–C_3_H_6_]^+^), followed by the loss of one molecule of C_2_H_2_, resulting in a secondary fragment at *m/z* 199.0603. The quasi-molecular ion of honokiol can further lose one molecule of CH_4_ and C_3_H_4_, generating fragments at *m/z* 251.0919 and *m/z* 211.0609, respectively. It can also lose one molecule of C_3_H_6_, producing a fragment ion at *m/z* 226.0840 ([M + H–C_3_H_6_]^+^). Another category is simple phenylpropanes. Using chlorogenic acid (C_16_H_18_O_9_) as the reference constituent, its quasi-molecular ion is m/z 355.102. Initially, the benzoyloxy group of phenylpropanoic acid breaks adjacent to the C atom, generating two possible fragment ions: *m/z* 163.0384 ([M + H–C_7_H_12_O_6_]^+^) and *m/z* 193.0503 ([M + H–C_9_H_6_O_3_]^+^). The former can undergo two possible fragmentation pathways: one is the removal of one molecule of water from the ortho-benzodihydroxy group, generating a fragment at *m/z* 145.0274, and the other is the consecutive loss of CO and H_2_O, resulting in secondary fragments at *m/z* 135.0434 and *m/z* 117.0329, respectively. These simple phenylpropane constituents produce characteristic ions of [M + H–C_7_H_12_O_6_]^+^, [M + H–C_7_H_12_O_6_–CO]^+^, and [M + H–C_7_H_12_O_6_–CO–H_2_O]^+^. MN1 and MN12 of the Dayuanyin decoction extract contained phenylpropane constituents. Information related to the constituents is shown in Table 2, and the fragmentation pathways of the constituents are shown in Figure 7.

### Unannotated analysis points of saponin constituents in MN


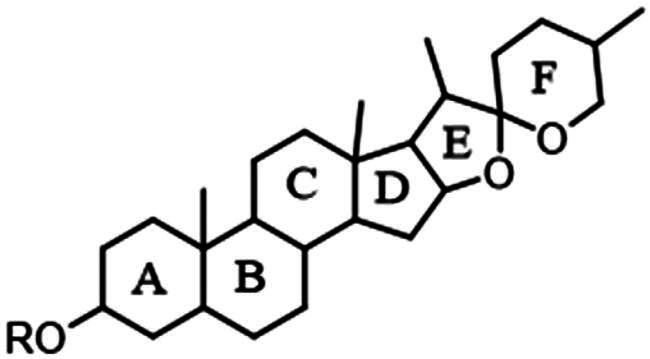


Basic structure of the steroid

**Figure 8.**
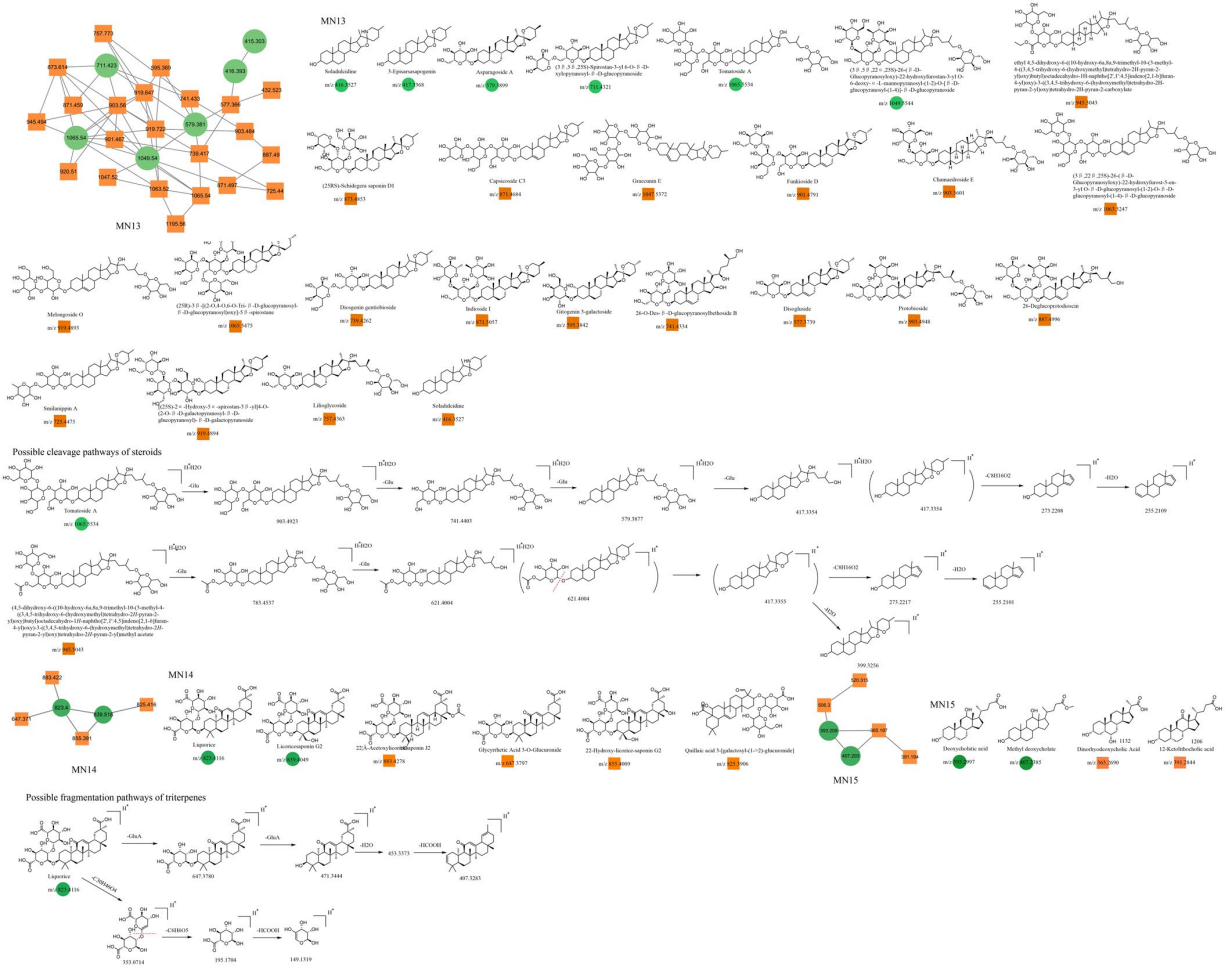
Structure of the MN13–MN15 constituents and the possible fragmentation pathway of representative constituents.

The saponin constituents in Dayuanyin decoction mainly included steroidal saponins and triterpenoid saponins, which are susceptible to losing sugar groups, sugar aldehyde groups, H_2_O, CH_3_, and side chain groups. These saponins are derived from *Anemarrhena asphodeloides* and *Glycyrrhiza uralensis.* Taking tomatoside A (C_51_H_86_O_24_) as an example for fragmentation analysis, it generates a dehydrated positive ion at *m/z* 1065.5534 ([M + H–H_2_O]^+^) in the positive ion mode. Subsequently, it sequentially loses four glucose molecules, resulting in *m/z* 903.4923 ([M + H–H_2_O–Glu]^+^), *m/z* 741.4403 ([M + H–H_2_O–2Glu]^+^), *m/z* 579.3877 ([M + H-H_2_O–3Glu]^+^), and *m/z* 417.3354 ([M + H–H_2_O–4Glu]^+^). Then, the oxygen-containing heterocycle is removed, resulting in a secondary fragment ion at *m/z* 417.3354 ([M + H-H_2_O–4Glu–C_8_H_16_O_2_]^+^). Further removal of one water molecule leads to a fragment at *m/z* 255.2109. The basic carbon scaffold of steroidal constituents is derived from spirostanes, with the F ring forming a cyclic or chain structure. The fragmentation pattern mainly involves the consecutive cleavage of glycosidic bonds on the A or F rings, as well as the loss of two oxygen-containing rings (–C_8_H_16_O_2_). For triterpenoid saponins, taking liquorice (C_42_H_62_O_16_) as an example for fragmentation analysis, the constituent exhibits a quasi-molecular ion at *m/z* 823.4116 in the positive ion mode. The fragmentation pattern of this constituent was similar to that of tomatoside A. It first loses two molecules of glucuronic acid consecutively, generating fragment ions at *m/z* 647.3780 ([M + H–GluA]^+^) and *m/z* 471.3444 ([M + H–2GluA]^+^). Then, the fragment ion loses one water molecule to produce a fragment at *m/z* 453.3373. Finally, the carboxyl group on the pentacyclic ring is cleaved, resulting in a secondary fragment ion at *m/z* 407.3283 ([M + H–2GluA–H_2_O–HCOOH]^+^). Additionally, *m/z* 353.0714 ([M + H–C_30_H_46_O_4_]^+^) is a fragment ion of tomatoside A obtained after the removal of the entire pentacyclic ring structure, leaving two glucuronic acid aldehyde groups. Subsequently, the removal of one molecule of glucuronic acid generates a fragment ion at *m/z* 195.1704. Finally, the carboxyl group is lost, resulting in a secondary fragment ion at *m/z* 149.1319 ([M + H–C_30_H_46_O_4_–C_6_H_6_O_5_–HCOOH]^+^). In this study, a possible new constituent of steroidal saponins was discovered, named 4,5-dihydroxy-6-((10-hydroxy-6a,8a,9-trimethyl-10-(3-methyl-4-((3,4,5-trihydroxy-6-(hydroxymethyl)tetrahydro-2H-pyran-2-yl)oxy)butyl)octadecahydro-1H-naphtho[2′,1′: 4,5]indeno[2,1-b]furan-4-yl)oxy)-3-((3,4,5-trihydroxy-6-(hydroxymethyl)tetrahydro-2H-pyran-2-yl)oxy)tetrahydro-2H-pyran-2-yl)methyl acetate. Its quasi-molecular ion was *m/z* 945.5043 ([M + H–H_2_O]^+^). Similar to tomatoside A, it consecutively lost two Glu molecules, resulting in a fragment ion at *m/z* 621.4004, which was 42 Da higher than the ion of tomatoside A (*m/z* 579.3877). Furthermore, combining the following secondary fragments at *m/z* 417, *m/z* 273, and *m/z* 255, which are the same as in tomatoside A, it can be inferred that there is likely a group of 42 Da connected to the glucose group in the structure of tomatoside A. It is speculated that the hydroxyl group on the glucose was esterified, forming a CH_3_COO- group. The fragmentation pathway of these constituents is illustrated in Figure 8. The triterpenoid saponin constituents identified in Dayuanyin decoction were predominantly the oleanane type, characterized by a relatively stable pentacyclic ring structure that is not easily cleaved. Fragmentation mainly involves the cleavage of the entire glucuronic acid group and the chemical bonds between glucuronic acid units. In this study, steroidal and triterpenoid constituents primarily originated from the MN13–MN15 constituent clusters. The chemical structures of these two constituent classes are depicted in Figure 8, and relevant information is found in Table 2.

### Unannotated analysis points of isoquinoline alkaloid constituents in MN

**Figure 9.**
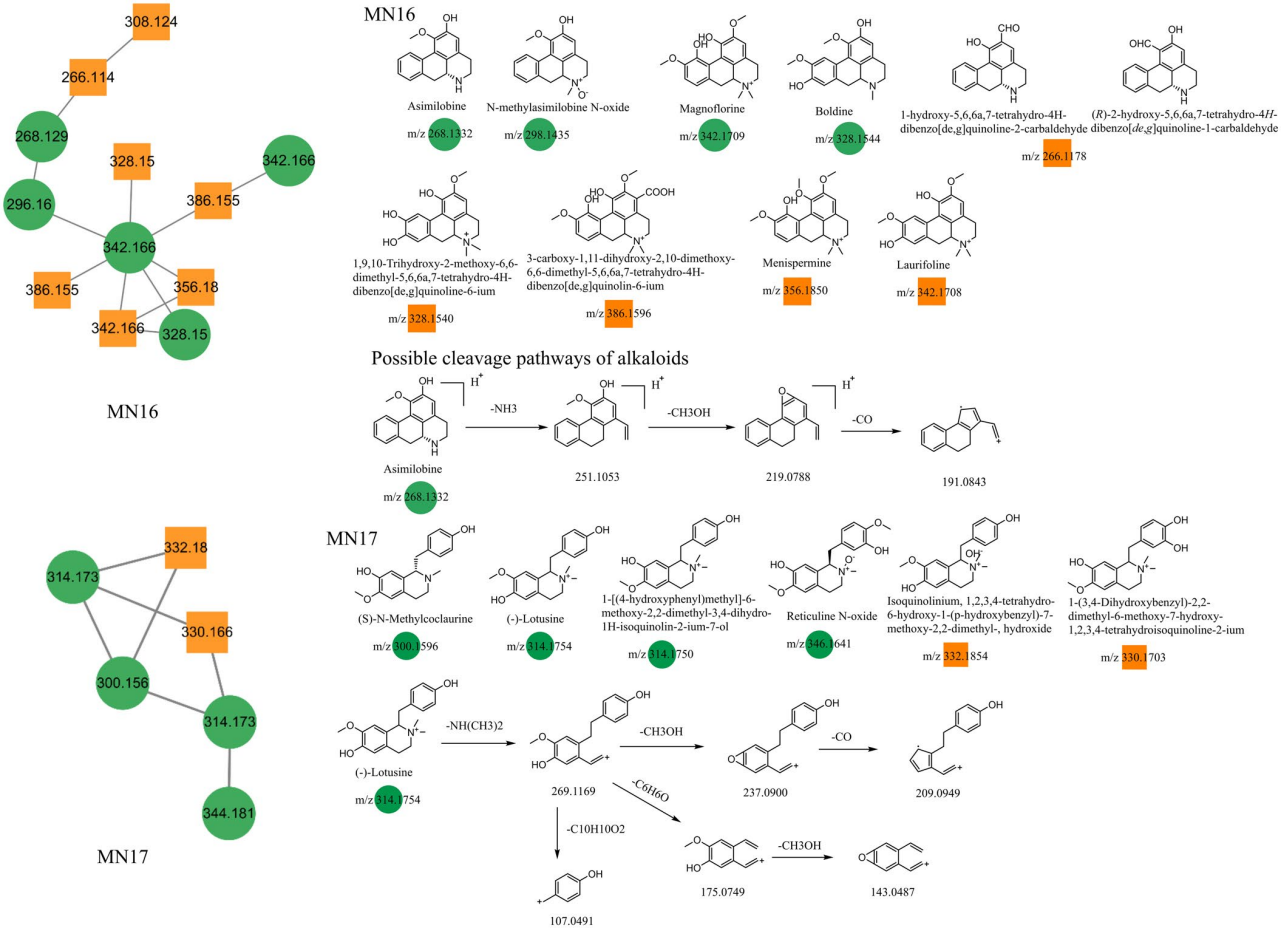
Structure of the MN16–MN17 constituents and the possible fragmentation pathway of representative constituents.

The alkaloids in Dayuanyin decoction were primarily identified in the positive ion mode, and their main sources were *Areca catechu* L. and *Magnoliae officinalis.* Tetrahydrobenzylisoquinoline alkaloids were predominantly present. Asimilobine and (–)-lotusine were used as examples for illustration purposes. Taking asimilobine as an example, its quasi-molecular ion peak in the positive ion mode is observed at *m/z* 268.1332. First, the N atom of the secondary amine breaks off from the adjacent two carbons, resulting in the loss of one molecule of NH_3_ and the generation of a fragment ion at *m/z* 251.1053 ([M + H–NH_3_]^+^). Then, the adjacent hydroxyl group (OH) and methoxy group (OCH_3_) on the phenyl ring detach one molecule of CH_3_OH, resulting in the formation of an oxygen-containing heterocyclic structure and a fragment ion at *m/z* 219.0788 ([M + H–NH_3_–CH_3_OH]^+^). Finally, the removal of one molecule of CO produces an MS/MS fragment ion at m/z 191.0843. (–)-Lotusine is a quaternary ammonium alkaloid, and it generates a quasi-molecular ion at *m/z* 314.1754 ([M]^+^) in the positive ion mode. It subsequently loses a dimethylamine group to form a fragment ion at *m/z* 269.1169. This ion can undergo multiple fragmentation pathways. First, it can sequentially lose a molecule of CH_3_OH and CO, resulting in fragment ions at *m/z* 237.0900 and *m/z* 209.0949 ([M–NH(CH_3_)_2_–CH_3_OH–CO]^+^), respectively. Second, ethyl group cleavage occurs between the two benzene rings, producing fragment ions at *m/z* 175.0749 ([M–NH(CH_3_)_2_–C_6_H_6_O]^+^) and *m/z* 107.0491 ([M–NH(CH_3_)_2_–C_10_H_10_O_2_]^+^). The former further eliminates a CH_3_OH molecule from the adjacent functional groups on the benzene ring, yielding a secondary fragment at *m/z* 143.0487. The main mass spectrometry fragmentation pathways of the alkaloid constituents in Dayuanyin decoction were the loss of N-containing groups, the condensation of adjacent O-containing groups on the benzene ring to form an O-containing ring, and the loss of CO. The possible new constituents in the alkaloid constituents had a molecular ion at *m/z* 266.1178, which could be either 1-hydroxy-5,6,6a,7-tetrahydro-4H-dibenzo[de,g]quinoline-2-carbaldehyde or 2-hydroxy-5,6,6a,7-tetrahydro-4H-dibenzo[de,g]quinoline-1-carbaldehyde. When compared to asimilobine, both had the same fragment ions at *m/z* 219 and 191. One of the fragment ions in the new constituents, at *m/z* 249, was 2 Da less than that of asimilobine, suggesting a reduced number of hydrogen atoms on the parent nucleus and an increased degree of unsaturation. Considering the difference between CH_2_OH and CHO, the position of CHO may differ in the two constituents. Although the secondary mass spectra of the two constituents were the same, the difference in the connection positions of CHO and CH_2_OH suggested the existence of two different constituents. The alkaloid constituents mainly belonged to the MN16 and MN17 clusters. The chemical structures of the relevant constituents are shown in Figure 9. Table 2 provides the related mass spectrometry information.

### Unannotated analysis points of N-containing constituents in MN

**Figure 10.**
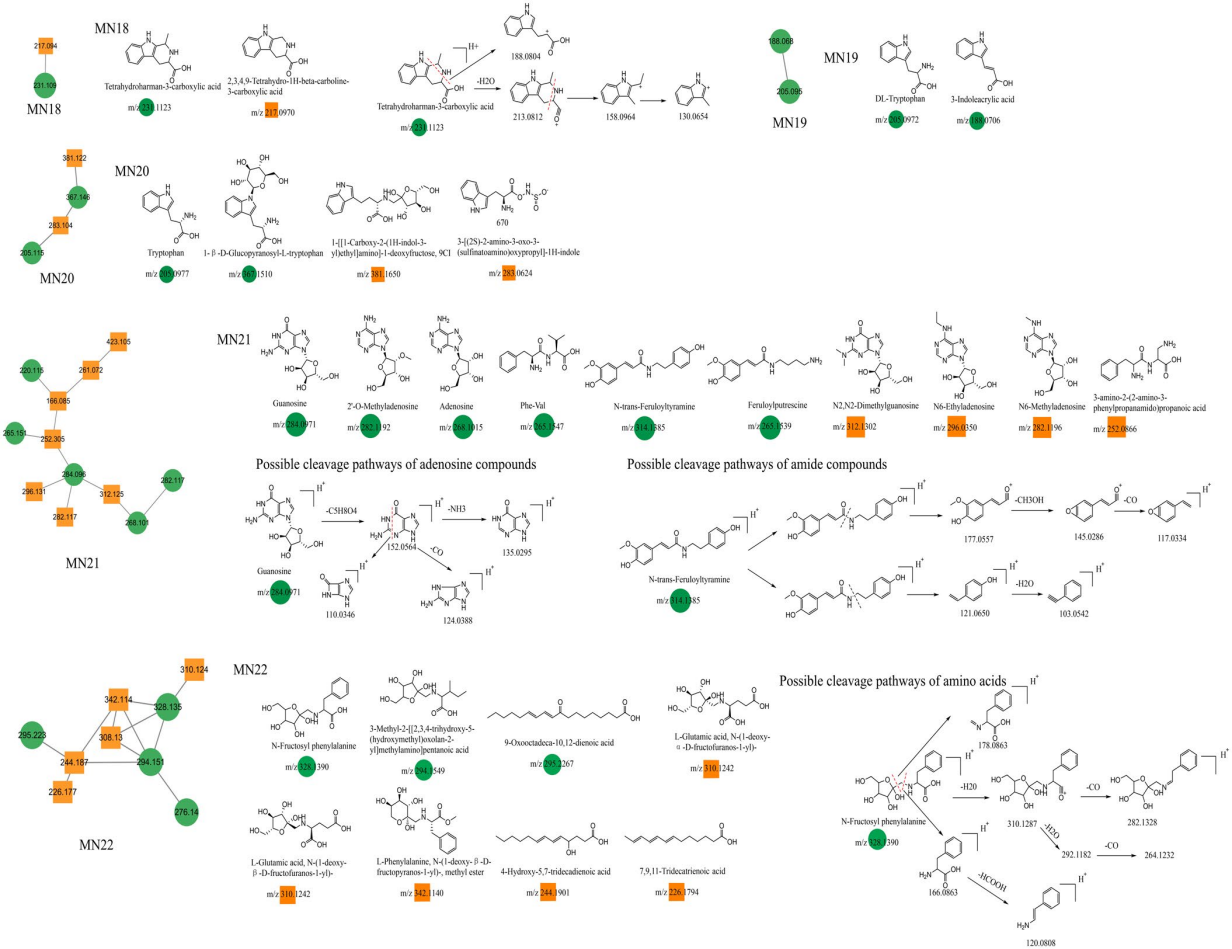
Structure of the MN18–MN22 constituents and the possible fragmentation pathway of a representative constituent.

This section mainly focuses on two types of *N*-containing constituents, including guanosine and amino acids. Taking guanosine, tetrahydroharman-3-carboxylic acid, and *N*-fructosyl phenylalanine as examples, guanosine exhibits a molecular ion peak at *m/z* 284.0971 ([M + H]^+^) in the positive ion mode. It first loses a pentose molecule, generating a fragment ion at *m/z* 152.0564. Subsequently, it undergoes various fragmentation pathways. The first pathway involves the simple loss of an NH_3_ molecule, resulting in a fragment ion at *m/z* 135.0295. The second pathway involves the cleavage of a six-membered ring containing nitrogen, leading to the elimination of an (NH_2_)_2_CH_2_ molecule. The remaining portion forms a cyclic lactam with four carbons, producing a fragment ion at *m/z* 110.0346. The third pathway involves the loss of CO from the six-membered ring, yielding a fragment ion at *m/z* 124.0388. This type of nucleoside has the same characteristic ions at *m/z* 135/110. Another adenine-based constituent, which is similar to guanosine in its basic core structure but differs by having an additional *N*-containing group in the six-membered ring and lacking a carbon-oxygen double-bond, exhibits common secondary ions at *m/z* 136/119. Tetrahydroharman-3-carboxylic acid and *N*-fructosyl phenylalanine were selected as reference constituents for the fragmentation patterns of amino acids in this study. Tetrahydroharman-3-carboxylic acid easily undergoes dehydration in the positive ion mode, producing a fragment ion at *m/z* 213.0812. Subsequently, further ring opening of the *N*-containing six-membered heterocyclic structure results in the formation of constituents with a fused benzopyrrole structure at *m/z* 158.0964. Finally, the loss of an ethylene molecule yields a fragment ion at *m/z* 130.0654. Another fragmentation pathway involves the direct cleavage of the *N*-containing six-membered heterocyclic structure on the precursor ion, resulting in a fragment ion at *m/z* 188.0804 ([M + H–NH = CHCH_3_]^+^). N-fructosyl phenylalanine (*m/z* 328.139, [M + H]^+^) exhibits three different fragmentation pathways. First, the constituents undergo cleavage of the pentose moiety, generating a fragment ion with a carbon-nitrogen double-bond at *m/z* 178.0863. Alternatively, consecutive losses of H_2_O and CO lead to the formation of secondary fragments at *m/z* 310, 282, 292, and 264. Carbon-nitrogen single-bond cleavage generates a fragment ion with an amino acid structure at *m/z* 166.0863 ([M + H–C_6_H_5_O_10_]^+^), followed by the loss of an HCOOH molecule, resulting in a fragment ion at *m/z* 120.0808. In this study, amino acid constituents were derived from MN18–MN22 constituent clusters, whereas adenine constituents originated from the MN21 cluster. The chemical structures of these constituents are shown in Figure 10, and relevant information is provided in Table 2.

### Unannotated analysis points of other constituents in MN

**Figure 11.**
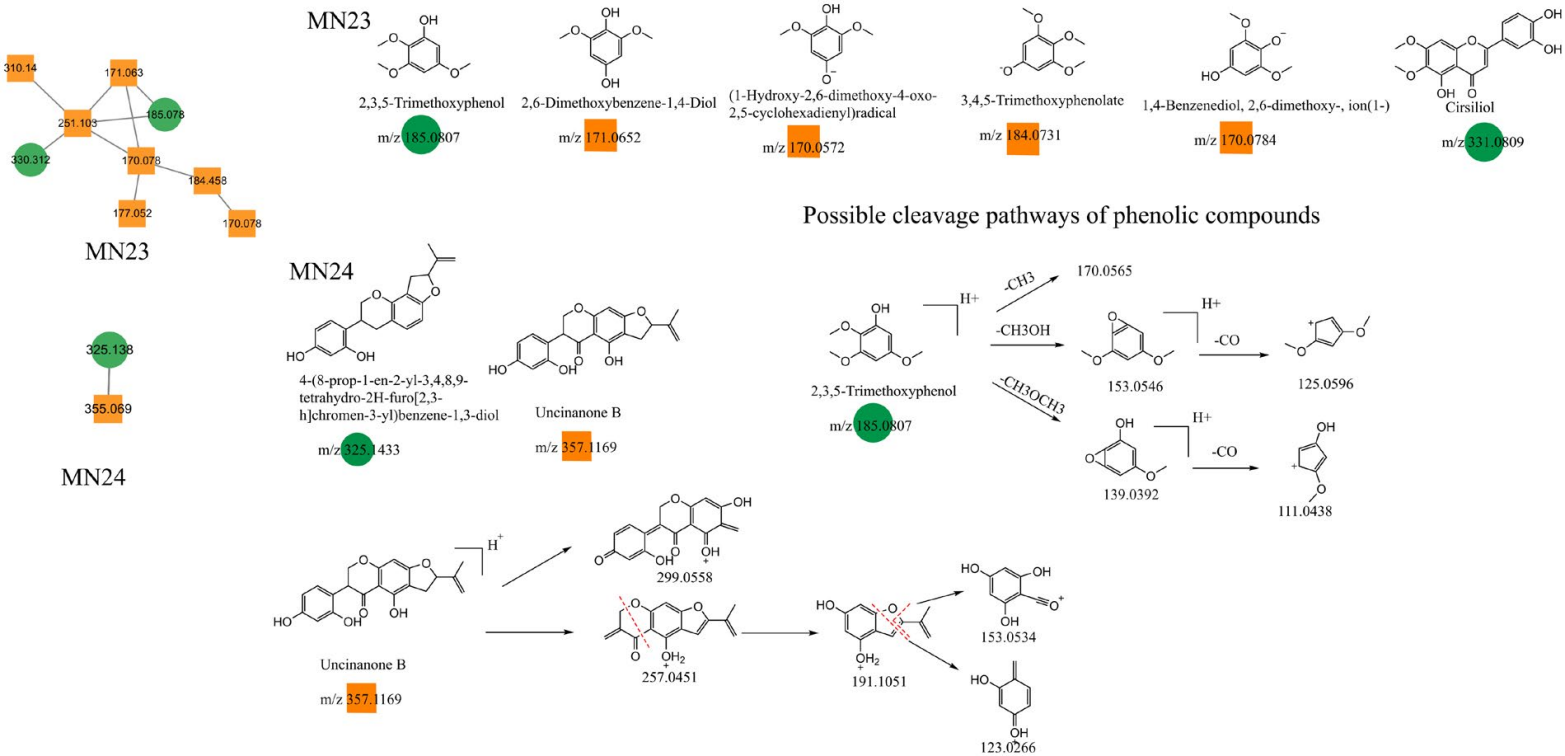
Structure of the MN23–MN24 constituents and the possible fragmentation pathway of representative constituents.

Other constituents identified in Dayuanyin decoction included phenols, phenolic glycosides, fatty acid chains, and other constituents. A few examples follow. 2,3,5-Trimethoxyphenol is a simple phenol constituent, and its quasi-molecular ion appears at *m/z* 185.0807 ([M + H]^+^) in the positive ion mode. It can lose a methyl group (CH_3_) to generate a fragment ion at *m/z* 170.0565. Alternatively, the condensation of adjacent OH and OCH_3_ groups on the benzene ring can occur, followed by the loss of a molecule of CH_3_OH, resulting in a fragment ion at *m/z* 153.0546. The cyclic structure containing oxygen loses a molecule of CO, generating a secondary fragment ion with a cyclopentadiene structure at *m/z* 125.0596 ([M + H–CH_3_OH–CO]^+^). Another fragmentation pathway is similar to the previous one, where two adjacent OCH_3_ groups on the benzene ring undergo condensation. The resulting cyclic structure with oxygen then loses a CO molecule, producing a fragment ion at *m/z* 111.0438 ([M + H–CH_3_OCH_3_–CO]^+^). In this study, other class constituents were mainly found in the MN1 and MN23–MN24 clusters. Specific information about the constituents appears in Table 2, and their structural formulas are shown in Figures 4 and 11.

The MN of the Dayuanyin decoction extract was generated using the GNPS method. A total of 74 clusters were generated from the extract, out of which 24 were analyzable. Thirty-six constituents were found to match constituents in the database. The 24 clusters from the Dayuanyin decoction extract were visualized using Cytoscape 3.9.2, as shown in Figure 2. In the figure, labeled nodes are represented by circles, while inferred unlabeled nodes are represented by squares. Each node represents a constituent, with the molecular weight of the parent ion indicated inside the node. In this study, magnoflorine, 3′-demethylnobiletin, and adenosine were inferred as unlabeled points in MN. Therefore, these three constituents were selected to verify the accuracy of MN inference using reference standards along with literature matching. The sample was then analyzed using the chromatographic conditions employed for the Dayuanyin decoction extract, and the observed secondary fragments were compared with the three inferred constituents to determine if they matched.

### Validation of MN results

**Figure 12.**
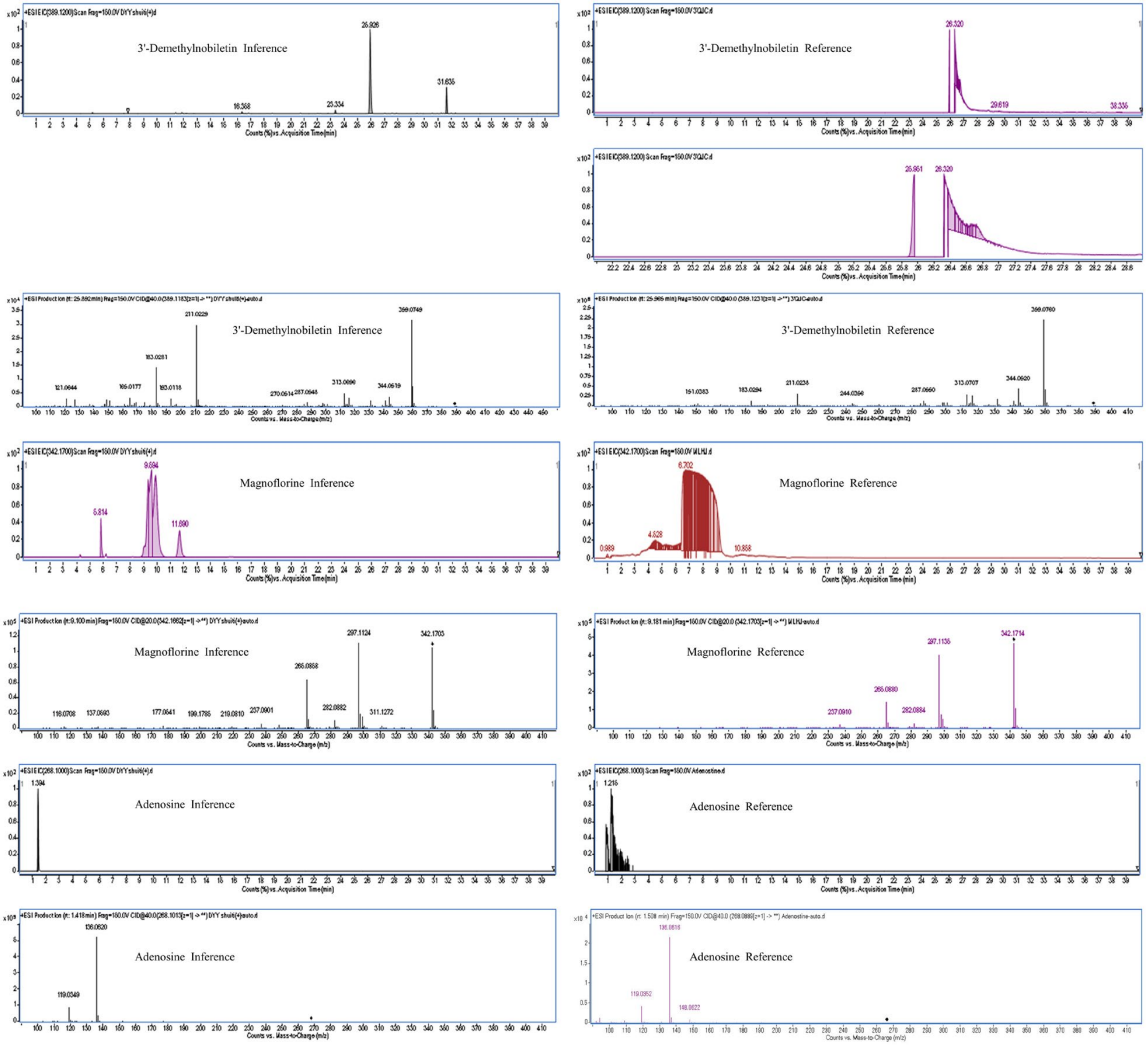
The extracted ion chromatogram and MS/MS spectrum of three constituents of the standard reference substance and the Dayuanyin decoction extract.

This study selected three unannotated nodes in MN belonging to the classes of flavonoids, adenosines, and alkaloids to ensure the correctness of the speculated constituents: 3′-demethylnobiletin, adenosine, and magnoflorine. These three constituents were chosen because they are representatives of different and abundant constituent types and are readily available for purchase. First, these three constituents were dissolved in methanol to prepare control solutions, each at a concentration of 1.0 mg/mL. Then, MS and MS/MS spectrometry data were obtained by UPLC-QTOF-MS analysis. The observed secondary fragments were compared with the three inferred constituents to determine if they matched. The results in Table 3 and Figure 12 demonstrate that the retention time and ion fragments of the reference substances matched those of the predicted constituents, confirming the accuracy of our method. We used MN analog clustering to identify constituents with similar fragmentation pathways.

**Table 3.** Mass spectrum/chromatographic information of the constituents in the standard reference substance and dayuanyin decoction extract.

Identification	Formula	[M + H]+	MS2	tR (min)	
3′-Demethylnobiletin	C_20_H_20_O_8_	389.1231	359.0749,344.0519,313.0698,211.0238,183.0289	25.926	Inference
3′-Demethylnobiletin	C_20_H_20_O_8_	389.1231	359.0760,344.0619,313.0707,211.0218,183.0294	25.961	Reference
Magnoflorine	C_20_H_24_NO_4_+	342.1709	297.1112,282.0823,265.0803,237.0901	9.100	Inference
Magnoflorine	C_20_H_24_NO_4_+	342.1709	297.1135,282.0884,265.0880,237.0910	9.181	Reference
Adenosine	C_10_H_13_N_5_O_4_	268.1015	136.0620,119.0349	1.394	Inference
Adenosine	C_10_H_13_N_5_O_4_	268.1015	136.0616,119.0352	1.518	Reference

## Discussion

The combination of UPLC-QTOF-MS and GNPS molecular networking was a fast and reliable method for analyzing the constituents of Dayuanyin decoction extract. This approach overcomes the high cost and low-efficiency issues associated with traditional methods for discovering constituents in traditional Chinese medicine and may promote the discovery of new constituents of traditional Chinese medicine. The identified unknown constituents can be validated using standard substances based on their retention time and fragment ions, thereby avoiding the complexities involved in the initial identification of novel constituents. This study identified a total of 216 constituents in Dayuanyin decoction. The main constituents were flavonoids, amino acids, alkaloids, triterpenes, steroidal saponins, phenylpropanoids, and other constituents, primarily attributed to the high polarity of constituents in aqueous extracts. Relatively fewer constituents from *Amommum tsao-ko* were found, which may have been due to the easy loss of its main active constituent, essential oil, during the extraction process and the limitations of UPLC-QTOF-MS. In the future, gas chromatography and other techniques can be used to study the volatile constituents of Dayuanyin decoction to improve research into its foundational components. However, his study also had certain limitations. The positive ion mode was chosen for analyzing the Dayuanyin decoction water extract. A comparison of the possible constituents under positive and negative ion modes found that the positive ion mode showed a wider variety of constituents and also covered the constituents found in the negative ion mode. However, some constituents may only be detected in negative ion mode and may not appear in positive ion mode. Thus, some constituents may have been missed in the positive ion mode analysis. Due to the limitations of the MN database, some constituents in certain clusters could not be identified because they lacked annotations. Additionally, several types of constituents may be present within the same constituent′s cluster, possibly due to partial similarities in their secondary fragments, leading them to be categorized under the same constituent′s cluster by default. Ultimately, the constituents identified in this study need to be further isolated for *in vitro* or *in vivo* activity screening.

## Conclusions

This study established a strategy based on UPLC-QTOF-MS combined with GNPS to analyze 216 constituents in Dayuanyin decoction extract successfully. Among them, 36 constituents were identified by matching against Agilent and Metlin databases, as well as a self-built database compiled from the literature and PubChem. One hundred and eighty constituents were identified from 24 clusters using MN provided by GNPS. These constituents included flavonoids, amino acids, alkaloids, triterpenes, steroidal saponins, phenylpropanoids, and other constituents. The fragmentation patterns of representative constituents of each type were also analyzed, providing a material basis and scientific evidence for the further development and utilization of Dayuanyin decoction.
